# A *pyrF*-Based Efficient Genetic Manipulation Platform in Acinetobacter baumannii To Explore the Vital DNA Components of Adaptive Immunity for I-F CRISPR-Cas

**DOI:** 10.1128/spectrum.01957-22

**Published:** 2022-09-01

**Authors:** Shuqi Wu, Run Xu, Mengjiao Su, Can Gao, Yang Liu, Yujia Chen, Guangxin Luan, Xu Jia, Rui Wang

**Affiliations:** a Non-coding RNA and Drug Discovery Key Laboratory of Sichuan Province, Chengdu Medical College, Chengdu, Sichuan, China; b CAS Key Laboratory of Microbial Physiological and Metabolic Engineering, Institute of Microbiology, Chinese Academy of Sciences, Beijing, China; c Yan’an Key Laboratory of Microbial Drug Innovation and Transformation, School of Basic Medicine, Yan’an University, Yan’an, Shaanxi, China; University of Hong Kong

**Keywords:** *Acinetobacter baumannii*, *pyrF* based, genetic manipulation system, CRISPR-Cas

## Abstract

Acinetobacter baumannii is an important pathogenic bacterium with multidrug resistance which causes infections with high mortality rates. In-depth genetic analysis of A. baumannii virulence and drug-resistant genes is highly desirable. In this study, we utilized the conserved *pyrF*-flanking fragment to rapidly generate uracil auxotrophy hosts with *pyrF* deleted in model and clinical A. baumannii strains and then introduced the *pyrF* gene as the selectable and counterselectable marker to establish a series of gene manipulation vectors. For gene deletion with the suicide *pyrF*-based plasmid, the second-crossover colonies screened with the *pyrF*/5-fluoroorotic acid (5-FOA) system were obtained more quickly and efficiently than those screened with the *sacB*/sucrose system. By using the replicative plasmid, the recognized protospacer-adjacent motif (PAM) bias for type I-F CRISPR was experimentally revealed in A. baumannii AYE. Interestingly, interference recognized only the PAM-CC sequence, whereas adaptation priming tolerates 4 PAM sequences. Furthermore, we also performed a rapid and extensive modification of the I-F CRISPR-Cas elements and revealed that the role of double-nucleotide sequence mutants at the end of the repeat could be critical during both CRISPR interference and priming; we also found strong biases for A and demonstrated that adaptation could tolerate certain sequence and size variations of the leader in A. baumannii. In conclusion, this *pyrF*-based genetic manipulation system was readily applicable and efficient for exploring the genetic characteristics of A. baumannii.

**IMPORTANCE** In this study, we developed the widely applicable and efficient *pyrF*-based selection and counterselection system in A. baumannii for gene manipulation. In most cases, this *pyrF*/5-FOA genetic manipulation system was very effective and enabled us to obtain marker-free mutants in a very short period of time. Utilizing this system and the separate mechanism of interference and/or primed adaptation, our experiments revealed some recognition mechanism differences for the key DNA elements of PAM, leader, and repeat in the priming adaptation process of the I-F CRISPR-Cas systems of A. baumannii, which provided some new and original insights for the study of the molecular mechanisms of these processes and laid a foundation for further studies.

## INTRODUCTION

Acinetobacter baumannii is a common and important pathogenic bacterium involved in hospital infections that causes wound infection, osteomyelitis, respiratory infections, bacteremia, etc. Since the 1980s, multidrug-resistant A. baumannii has become a challenge in the treatment of nosocomial infections; thus, the drug resistance mechanisms of A. baumannii have drawn constant attention. Over the last decade, the resistance rate of A. baumannii has been increasing, and strains resistant to carbapenems have been widely reported (1–4). Tigecycline and colistin were once considered the last antibiotics used to treat carbapenem-resistant A. baumannii (5, 6), but unfortunately, A. baumannii strains resistant to tigecycline and colistin have also appeared recently (7, 8). The virulence mechanisms of A. baumannii have also been a hot topic in recent years, as high mortality rates are observed in those infected in the hospital environment (9). Therefore, understanding the molecular mechanisms of drug resistance, cytotoxicity, and virulence of multidrug-resistant A. baumannii is crucial.

Uncovering the mechanisms of A. baumannii drug resistance and virulence requires large amounts of genetic analysis. Previously, the lack of genetic manipulation tools and methods for studying these issues hindered the understanding of the diseases caused by A. baumannii. In the last several years, various putative methods for transforming exogenous DNA into A. baumannii have been developed and optimized, such as conjugation, electrotransformation, and natural transformation via motility (10–13). Recombinant cloning technology is a quick and efficient way to perform genetic manipulation *in vivo* which usually uses plasmids with antibiotic selection markers transformed into A. baumannii to create mutants (14). However, that brings antibiotic resistance genes into the genome, and repeated use of antibiotics for stress screening also causes the emergence of resistant colonies, so this method is not suited for multiple gene deletions. For multidrug-resistant A. baumannii strains, it was usually not easy to find a suitable antibiotic marker. To help address this problem, hygromycin B, which is an aminoglycoside antibiotic not used to treat infections in humans and has antimicrobial activity against a wide range of microorganisms, was applied to a diverse set of vectors (15). Later, Amin et al. demonstrated that a non-antibiotic resistance marker could be broadly used in most A. baumannii strains and developed the pMo130Tel^R^ vector, containing a tellurite resistance gene (16). In this study, we used the pMo130Tel^R^ vector as the selection marker to broadly generate A. baumannii hosts with *pyrF* deleted. This method does not use antibiotic selection for the gene deletion mutants and is beneficial for generating multiple gene deletions in a single strain. Recombination-mediated genetic engineering is one of the common methods to generate A. baumannii mutants. Usually, a second recombination event is initiated by the *sacB* gene as a counterselection cassette. The *sacB* gene expresses levansucrase in the presence of sucrose and is lethal in some Gram-negative bacteria, and this system was the most used in A. baumannii. However, when we used vectors with the *sacB*/sucrose system in A. baumannii, it always worked inefficiently and required a long time to subculture with sucrose for counterselection (15–17). This may be due to problems with the expression of the *sacB* gene or the function of levansucrase in A. baumannii. Tucker et al. (14) developed the recombinase-catalyzed homologous recombination method. This method can delete genes by a single-step transformation, but this brings antibiotic resistance genes into the genome, and the plasmids (RecAb-carrying and FLP-carrying plasmids) were both transformed and naturally removed twice, so there was a significant time cost (14).

Researchers found that another type of non-antibiotic resistance selectable marker, i.e., auxotrophic markers such as amino acid auxotrophy, NADPH auxotrophy, thymidine auxotrophy, and uracil auxotrophy, is also a useful selectable tool in gene editing and vaccine production (17–21). Uracil auxotrophy has been successfully constructed in multiple strains, and some related genes are also widely used, such as *pyrF*, URA3, etc. (19, 22–24). The *pyrF* gene encodes orotidine-5′-monophosphate decarboxylase (25), which can convert orotidine-5′-monophosphate into UMP as a key intermediate for synthesis of uracil nucleotides (26), so it was widely used as a selectable marker in uracil auxotrophy *pyrF* mutants with medium lacking uracil. 5-Fluoroorotic acid (5-FOA) is an analogue of orotic acid which can be converted to a highly toxic compound (5-fluoro-UMP) by the product of *pyrF* and leads to bacterial death due to toxicity (24). Therefore, if the *pyrF* gene is deleted, the function of autonomously synthesizing uracil in the bacteria will be lost, and the mutants will be resistant to 5-FOA. Therefore, the *pyrF* gene was widely used as a selection and counterselection marker in uracil auxotrophy *pyrF* deletion mutants for gene manipulation (27–30). Moreover, to our knowledge, there are no previous reports of applying the *pyrF* gene as a selection and counterselection marker in A. baumannii.

Clustered regularly interspaced short palindromic repeats (CRISPRs) and CRISPR-associated (Cas) proteins constitute an adaptive immune system against invading genetic elements in prokaryotes (31, 32). Different systems are currently divided into six types (I to VI), comprising several subtypes, which are mainly distinguished based on the existence of Cas genes and the proteins they encode. Thirty-seven percent of Acinetobacter genomes encode type I-F CRISPR-Cas systems, with some of the largest CRISPR arrays found so far in bacteria (33–35). The CRISPR-Cas immunity system in type I-F functions in three steps: CRISPR adaptation always needs a priming process to recognize the invader DNA and then takes up the target (protospacer) to integrate into the CRISPR array; in CRISPR expression, the CRISPR array is transcribed and processed by Cas6 proteins, and in CRISPR interference, the CRISPR RNAs (crRNAs) guide the Cascade (CRISPR-associated complex for antiviral defense) complex to recognize the protospacer and protospacer-adjacent motif (PAM) and then neutralize the invaders. The recognition is achieved through complementary interaction between the crRNA spacer and the protospacer and is also dependent on a specific short PAM (36, 37). CRISPR immunity makes it possible to effectively distinguish spacer sequences in the host CRISPR locus and identical protospacer sequences in the invading DNA, so as to avoid self-targeting (38–40). Therefore, a discrimination mechanism is required in the interference and adaptation process; otherwise, CRISPR DNA itself may become a potential target in the interference and adaptation process (41, 42). In type III-A systems, the “self versus nonself” discrimination mechanism identifies spacer DNA as a “self” component by recognizing the sequence matching between the repeat sequence present at the 5′ handle of crRNA and the repeat sequence in CRISPR locus, and other target DNA that does not match the sequence is identified as “nonself” (43). In the Haloarcula hispanica type I-B system, the primed adaptation, which attaches to and cooperates with the interference pathway, distinguishes target from nontarget by CRISPR RNA guidance and PAM recognition (39). These previous data demonstrated that the PAM and repeat end nucleotides preceding a spacer DNA were closely related to the discrimination mechanism (39, 43). Here, we systematically mutated PAM sequences and CRISPR repeat sequences at positions −1 and −2 and detected the effects of 16 PAM and repeat mutations for interference and priming of the adaptation process of the I-F system.

The leader is also essential for adaptation, with different vital element functions. The leader sequence contains the promoter necessary to drive the transcription and encodes sequences recognized by the Cas1-2 complex and other cytokines, and importantly, it is required for insertion of repeat spacer units at the proximal leader edge of the repeat cassette (44). Previous studies that established the importance of leader sequences in the integration process point to the functional significance of the conserved DNA motifs (44–47). Therefore, it is necessary to explore the contributions and inherent specificity of CRISPR leader sequences in the adaptative immunity process.

Here, based on the pMo130Tel^R^ plasmid, we used 5-FOA as the counterselectable agent and utilized the conserved *pyrF* flanking fragment to rapidly generate the uracil auxotrophy *pyrF* deletion hosts in model and clinical A. baumannii strains. Then, the *pyrF* gene of A. baumannii AYE was introduced as the selectable and counterselectable marker to establish a series of gene manipulation vectors. Utilizing *pyrF*-based suicide plasmids, we modified the I-F CRISPR-Cas system, and second-crossover colonies screened by *pyrF*/5-FOA system were obtained more quickly and efficiently than those screened with the *sacB*/sucrose system. Utilizing the *pyrF*-based replicative plasmid, we also detected the adaptation immunity of the I-F CRISPR-Cas system, highlighting the importance of the Cas gene and CRISPR DNA element, and the PAM sequences recognized in the priming and interference process. Based on the above-described tools, we constructed AYEΔF-PA with two separate CRISPR variants, namely, priming CRISPR (pCRISPR) and adaptation CRISPR (aCRISPR), to produce the priming crRNAs and to accept new spacers, respectively (47), and eventually generated a set of repeat end mutants and leader mutants for exploring the vital DNA elements for priming, spacer integration, and interference.

## RESULTS

### Use of the conserved *pyrF* flanking fragment to rapidly generate uracil auxotrophy hosts in model and clinical A. baumannii strains.

**FIG 1:**
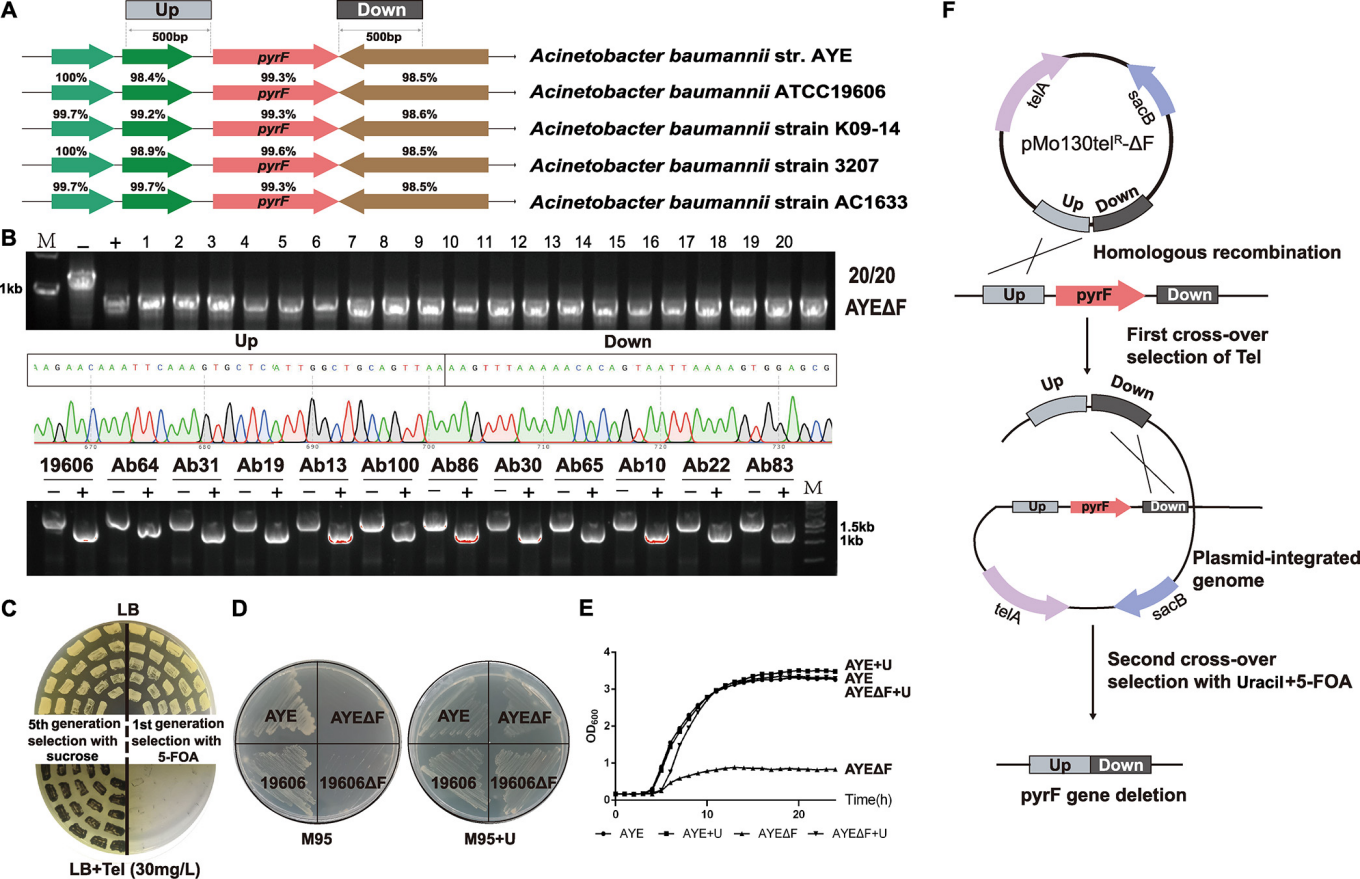
Use of the conserved *pyrF* flanking fragment to quickly generate the uracil auxotrophy hosts in model and clinical A. baumannii strains. (A) The *pyrF* gene and its flanking DNA sequences are highly conserved in A. baumannii. The 500-bp upstream (Up) and downstream (Down) regions flanking the *pyrF* genes were cloned into the suicide vector pMo130Tel^R^. (B) Twenty colonies were picked from first-generation screening with 5-FOA, and PCR amplification confirmed that these colonies all had the *pyrF* gene deleted. The efficiency was 20/20, confirmed by DNA sequencing. PCR results show that the *pyrF* genes of 11 clinical strains also were successfully deleted. −, wild-type strains; +, *pyrF* deletion mutants; M, marker. (C) Replica plate for colony screening. Twenty colonies were streaked onto replica plates after the fifth generation using the *sacB*/sucrose system and the first generation using the *pyrF*/5-FOA system. The second-crossover colonies were screened with sucrose or 5-FOA. After the first-generation screening, we found that it was easy to create a clean marker-free Δ*pyrF* mutant with the *pyrF*/5-FOA system. In contrast, the plasmid was difficult to eliminate from the genome with the *sacB*/sucrose system even five generations later. (D) Wild-type AYE and 19606 and mutant AYEΔF and 19606ΔF cells were streaked on M95 plates without or with uracil and incubated overnight. M95 plates were used to detect the uracil-auxotrophic Δ*pyrF* mutants. (E) Growth curve of the AYE and AYEΔF strains in different media (LB and LB+U). (F) By homologous recombination between pMo130Tel^R^-ΔF and the genome, a plasmid-integrated recombinant was obtained. A second homologous recombination between two homologous regions of the genome yielded the *pyrF* deletion (Δ*pyrF*) mutant. The second-crossover colonies were screened with 5-FOA. *telA* is the tellurite resistance marker in E. coli and A. baumannii.

One purpose of this study was to develop a widely applicable *pyrF*-based selection and counterselection system in A. baumannii for high-efficiency genetic manipulation. As an initial step, we identified *pyrF* genes and their adjacent sequences with the Basic Local Alignment Search Tool (BLAST) algorithm. The only *pyrF* gene in each genome was predicted to encode orotidine 5′-phosphate decarboxylase and may be responsible for uracil biosynthesis and 5-FOA sensitivity. The *pyrF* genes and their adjacent genes are highly conserved, with identity at or above 98% in many A. baumannii strains, such as A. baumannii AYE, A. baumannii ATCC 19606, A. baumannii strain K09-14, A. baumannii strain 3207, and A. baumannii strain AC1633 (Fig. 1A), suggesting that these highly conserved up- and downstream flanking sequences may be used for homologous recombination to widely generate *pyrF* deletion hosts in A. baumannii.

We chose to begin with the clinical model strain A. baumannii AYE because of its wide use, well-annotated genome sequence, and typical I-F CRISPR-Cas system. All strains and plasmids used in this study are listed in Table S1 in the supplemental material; 500-bp DNA sequences flanking *pyrF* were utilized for constructing pMo130Tel^R^-ΔF (Fig. 1A and F). pMo130Tel^R^-ΔF was integrated into the genome by first-crossover homologous recombination utilizing tellurite resistance as a selectable marker. The second-crossover colonies were screened with 5-FOA. After the first-generation screening, it was easy to create the Δ*pyrF* mutant by using the *pyrF*/5-FOA system, with an efficiency of 20/20 (Fig. 1B). PCR and DNA sequencing were then completed to confirm the *pyrF* gene deletion. With sucrose as a comparison, as described by Amin et al. (16), the plasmid was difficult to eliminate from the genome by the *sacB*/sucrose system, even after five generations (Fig. 1C).

In LB medium, the growth of AYEΔF was significantly inhibited compared with that of A. baumannii AYE, whereas it grew as well as AYE with uracil added (Fig. 1D). In the uracil-auxotrophic medium M95, it could not grow, whereas it grew well when uracil was supplied. These results indicated that the deletion of *pyrF* gene limits the synthesis of uracil and subsequently affects growth, but growth could be complemented by the addition of uracil. The growth of the Δ*pyrF* strain was uninhibited in LB plates containing uracil and 5-FOA, but the wild-type strain could not grow (Fig. S1), which means that the Δ*pyrF* strain was resistant to 5-FOA. These results suggested that the *pyrF* gene was responsible for the uracil biosynthesis and 5-FOA sensitivity in A. baumannii AYE.

To explore wider applications, we also easily deleted the *pyrF* gene in the A. baumannii model strains AYE and ATCC 19606 and 11 clinical strains by using the same method and same vector (pMo130Tel^R^-ΔF) (Fig. 1B). As uracil-auxotrophic hosts, these Δ*pyrF* mutants also could not grow in synthetic M95 medium lacking uracil, whereas the growth of wild-type strains was uninhibited. When M95 plates had enough uracil added, the mutants also grew well (Fig. 1E). As mentioned above, the *pyrF* gene was expected to be broadly useful as a selection and counterselection marker for the genetic manipulation system in A. baumannii. The uracil auxotrophy and 5-FOA susceptibility were expected to be utilized for the selection (first crossover) and counterselection (second crossover), respectively.

### Introduction of the *pyrF* gene to establish a series of gene manipulation vectors.

**FIG 2:**
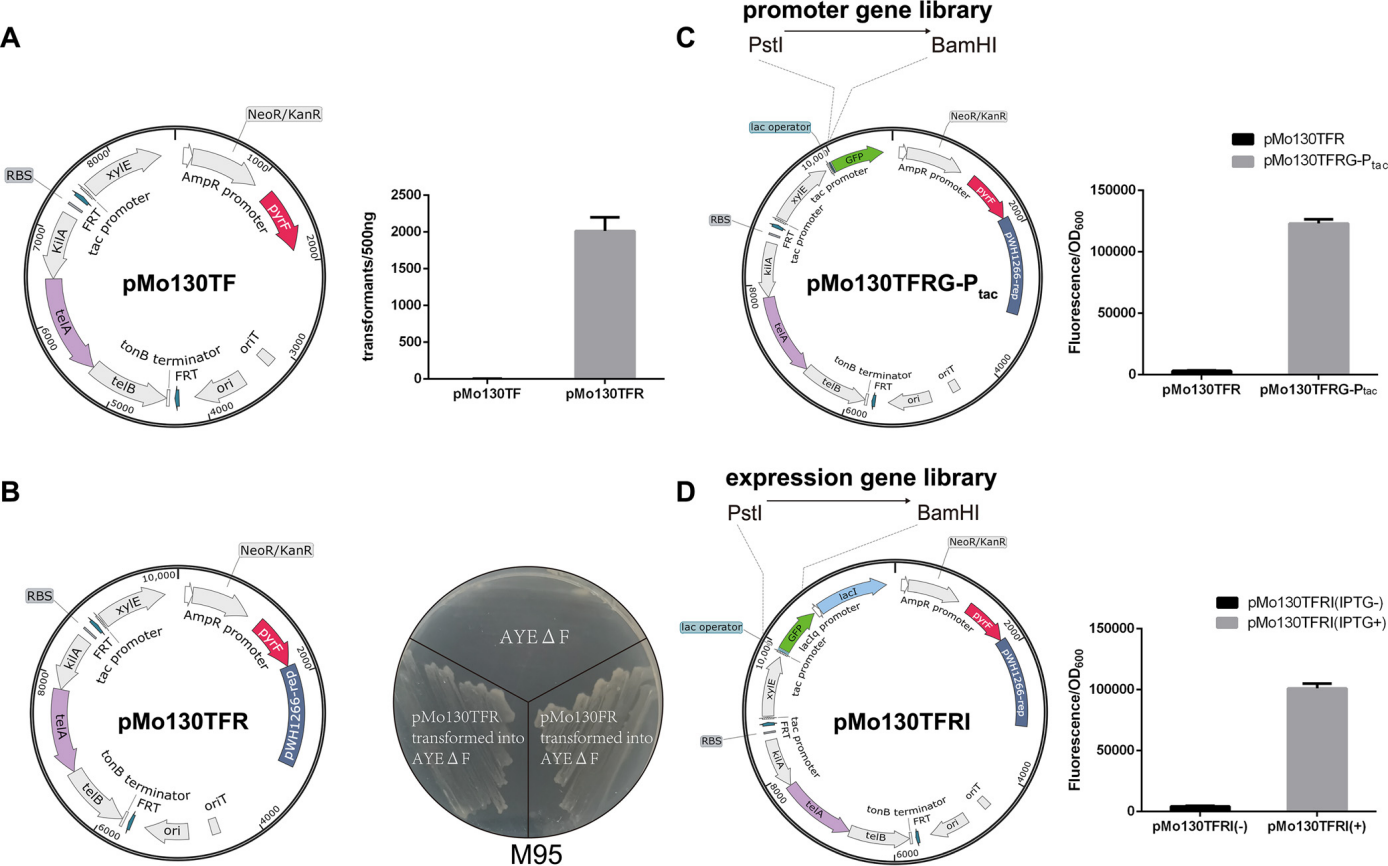
Introduction of the *pyrF* gene to establish a series of gene manipulation vectors. (A) The *pyrF* gene (along with its 200-bp upstream promoter sequence) was introduced into pMo130Tel^R^ to replace the *sacB* gene to generate pMo130TF. pMo130TF and pMo130TFR were each transformed into AYEΔF in triplicate. The transformation efficiency is shown. (B) The *rep* gene of pWH1266 was introduced into pMo130TF to generate pMo130TFR. pMo130TFR and pMo130FR transformants all grew well on the M95 plates. (C) P*_tac_* as a promoter example (this includes sequences controlling IPTG induction at the position of the *lacI* junction) and the GFP-mut3 gene with a strong bacterial RBS (AAAGAGGAGAAA) were introduced into pMo130TFR to generate pMo130TFRG-P_tac_. The promoter gene library was constructed in the BamHI/PstI site before RBS-GFP to detect the promoter function. Other researchers can change P_tac_ to different promoter genes according to their experimental purpose when using this plasmid. pMo130TFR and pMo130TFRG-P_tac_ were each transformed into AYEΔF in triplicate. The transformants were cultured in LB broth, and the fluorescence/OD_600_ values were measured (values are means and standard deviations [SD]). (D) The LacI gene and lac operator were introduced into pMo130TFRG-P_tac_ to generate pMo130TFRI. GFP-mut3 as a gene example was inserted into the BamHI/PstI site after the RBS. The expression gene library was constructed in the BamHI/PstI site to detect the expression function. Other researchers can change GFP-mut3 to different expression genes according to their experimental purpose when using this plasmid. pMo130TFRI was transformed into AYEΔF in triplicate. The transformants were cultured in LB broth without or with IPTG, and the fluorescence/OD_600_ values were measured (values are means and SD).

For developing a *pyrF*-based positive selection and counterselection system, the *pyrF*-carrying plasmids were also needed as vector tools. The *pyrF* gene along with its 200-bp upstream promoter sequence was first introduced into pMo130Tel^R^ or pMo130 to generate pMo130TF or pMo130F (lacking the tellurite resistance marker), respectively, by replacing the *sacB* gene (Fig. 2A; Fig. S2A). pMo130TF and pMo130F are suicide plasmids for targeted gene deletion and insertion by homologous recombination. The A. baumannii Δ*pyrF* transformants with pMo130TF/pMo130F derivatives integrated into the genome can be positively selected by plating the cells on Tel/Km-containing medium or on synthetic medium without uracil, such as M95. On the other hand, cells in which the pMo130TF/pMo130F-derived sequence is eliminated from the genome can be counterselected by using medium containing 5-FOA.

For gene expression, the autonomous replication plasmids pMo130TFR and pMo130FR were generated by introducing the *rep* gene of pWH1266 into pMo130TF and pMo130F, respectively (Fig. 2B; Fig. S2A). The transformation efficiency of pMo130TFR and pMo130FR increased sharply compared with that of pMo130TF and pMo130F in AYEΔF strains (Fig. 2A; Fig. S2B), suggesting that pMo130TFR and pMo130FR can replicate without integrating to the chromosome, while pMo130TF and pMo130F cannot replicate without the homologous sequence. The pMo130TFR and pMo130FR transformants all grew well on the M95 plates (Fig. 2B), which indicated that the *pyrF* gene in these plasmids could complete the uracil synthetic function and also can be used for positive selection in uracil-auxotrophic hosts. Therefore, these replicative plasmids can be autonomously replicated without integrating into the chromosome for gene expression, can be maintained by *pyrF*-based positive selection and may be eliminated by using medium containing 5-FOA as needed.

Based on the autonomous replication plasmid pMo130TFR, green fluorescent protein (GFP) reporter plasmids and isopropyl-β-d-thiogalactopyranoside (IPTG)-induced expression plasmids were also derived. For the GFP reporter plasmid pMo130TFRG, the GFP-mut3 gene with a strong bacterial ribosome binding site (RBS), AAAGAGGAGAAA (48), was introduced, and P*_tac_* as a promoter example (other researchers can change GFP-mut3 to different expression genes according to their experimental purpose when using this plasmid) was inserted into BamHI/PstI site before RBS-GFP to detect the promoter function. As shown in Fig. 2C, AYEΔF containing the plasmid pMo130TFRG-P_tac_ has a high ratio of fluorescence to optical density at 600 nm (OD_600_), whereas a very low ratio of fluorescence to OD_600_ was detected for the control without the promoter. This indicates that the GFP-mut3 gene was well expressed by the *tac* promoter and the GFP reporter plasmid works well. For the IPTG-induced expression plasmid pMo130TFRI, the LacI gene, P*_tac_*, lac operator, and the RBS were introduced and GFP-mut3 as a gene example was inserted into BamHI/PstI site after the RBS. The IPTG-induced expression plasmid also worked well, and the GFP-mut3 was well repressed by LacI and also well induced by IPTG (Fig. 2D). Other researchers can change P_tac_ and GFP-mut3 genes to different promoter and expression genes according to their experimental purpose when using these plasmids.

Therefore, these *pyrF*-based plasmids possessed a great capacity for gene manipulation as exceedingly convenient and versatile tools, with the *pyrF* gene as a selectable or counterselectable marker. The plasmids pMo130F and pMo130FR, with smaller sizes and greater loading capacity, may be used to insert or express large gene clusters. Selection of *pyrF* as the non-antibiotic resistance selection marker also could be a new choice.

### Use of the *pyrF*-based suicide plasmids for quick gene deletion in the I-F CRISPR-Cas system.

**FIG 3:**
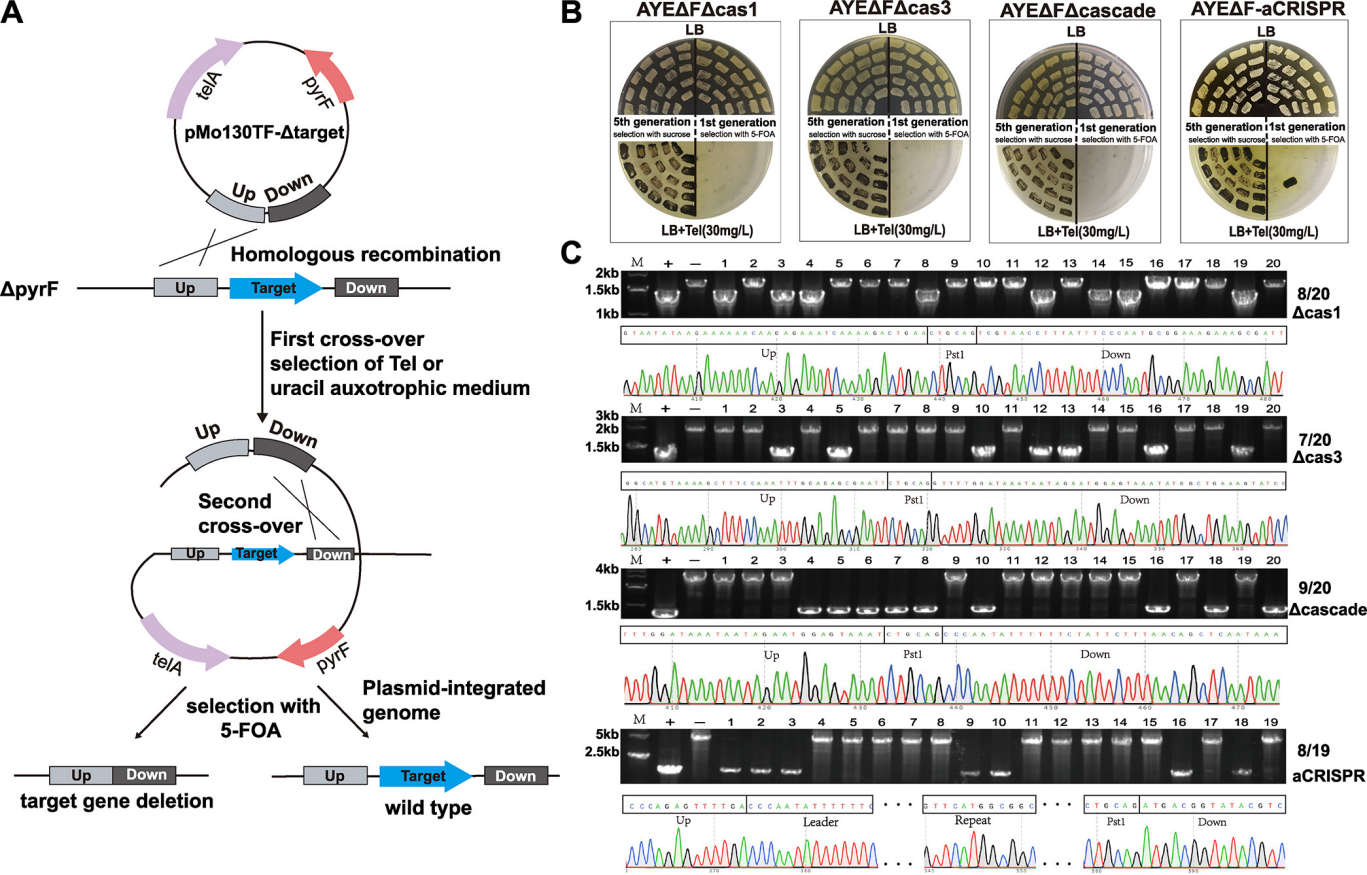
Use of the *pyrF*-based suicide plasmids for quick gene deletion of the I-F CRISPR-Cas system. (A) The target gene was deleted by the *pyrF*-based counterselectable system. The suicide plasmid pMo130TF carrying the *pyrF* gene and the flanking region of the target gene was transferred into the *pyrF* deletion mutants. The 5-FOA as the counterselection agent facilitated a second homologous recombination between the chromosome and vector. (B) Replica plates for screening the AYEΔFΔCas1, AYEΔFΔCas3, AYEΔFΔCascade, and AYEΔF-aCRISPR colonies. Twenty colonies were streaked onto the replica plates after the fifth generation using the *sacB*/sucrose system and after the first generation using the *pyrF*/5-FOA system. The second-crossover colonies were screened with 5-FOA or sucrose. For the *pyrF*/5-FOA system, the colonies with the plasmid-derived sequence removed were efficiency screened after the first generation, and almost all showed removal, with the efficiencies of 20/20, 20/20, 20/20, and 19/20. (C) Twenty colonies were picked from first-generation screening with 5-FOA, and PCR amplification confirmed that these colonies had deletions of Cas1, Cas3, Cascade, CRISPR (except the leader and a single repeat structure) target genes. The target genes were deleted with efficiencies of 8/20, 7/20, 9/20, and 8/19, as confirmed by DNA sequencing. −, wild-type strains; +, target deletion mutants; M, marker.

The *pyrF*-carrying suicide plasmid and corresponding *pyrF* deletion-containing uracil-auxotrophic host constitute a new gene knockout system for A. baumannii. In order to demonstrate the convenience and usefulness of this system, we utilized it to modify the Cas gene and CRISPR sequence of the I-F CRISPR-Cas system in A. baumannii AYEΔF (Fig. S3B). pMo130TF-ΔCas1, pMo130TF-ΔCas3, pMo130TF-ΔCascade, and pMo130TF-aCRISPR were generated to delete Cas1, Cas3, Cascade, and a large part of the CRISPR except the leader and a single repeat structure (aCRISPR), as described above. These vectors were then transformed into AYEΔF, and the transformants integrated into the genome were positively selected by M95 or LB plates with Tel (30 mg/L). 5-FOA (50 mg/L) was then supplied as the counterselection for subsequent incubation, and 20 colonies were picked to streak onto replica plates for detection of the efficiency of excision of the plasmid-derived sequence from the genome by homologous recombination. For the *pyrF*/5-FOA system, the colonies with the plasmid-derived sequence removed were efficiency screened after the first generation, and as expected, almost all had the sequences removed, with efficiencies of 20/20, 20/20, 20/20, and 19/20 (Fig. 3B). PCR and DNA sequencing were also carried out to confirm the results, and the efficiencies of deleting Cas1, Cas3, Cascade, and a large part of CRISPR were 8/20, 7/20, 9/20, and 8/19 (Fig. 3C).

As a comparison, we also utilized pMo130Tel^R^ (*sacB*/sucrose) to generate pMo130Tel^R^-ΔCas1, pMo130Tel^R^-ΔCas3, pMo130Tel^R^-ΔCascade, and pMo130Tel^R^-aCRISPR. However, it was difficult to isolate the mutant under selection with sucrose even after five generations (Fig. 1C and 3B). Therefore, the *pyrF*-based selection and counterselection system is an efficient and convenient choice for gene manipulation.

### Utilizing the *pyrF*-based replicative plasmid for detecting the adaptation immunity of the I-F CRISPR-Cas system.

**FIG 4:**
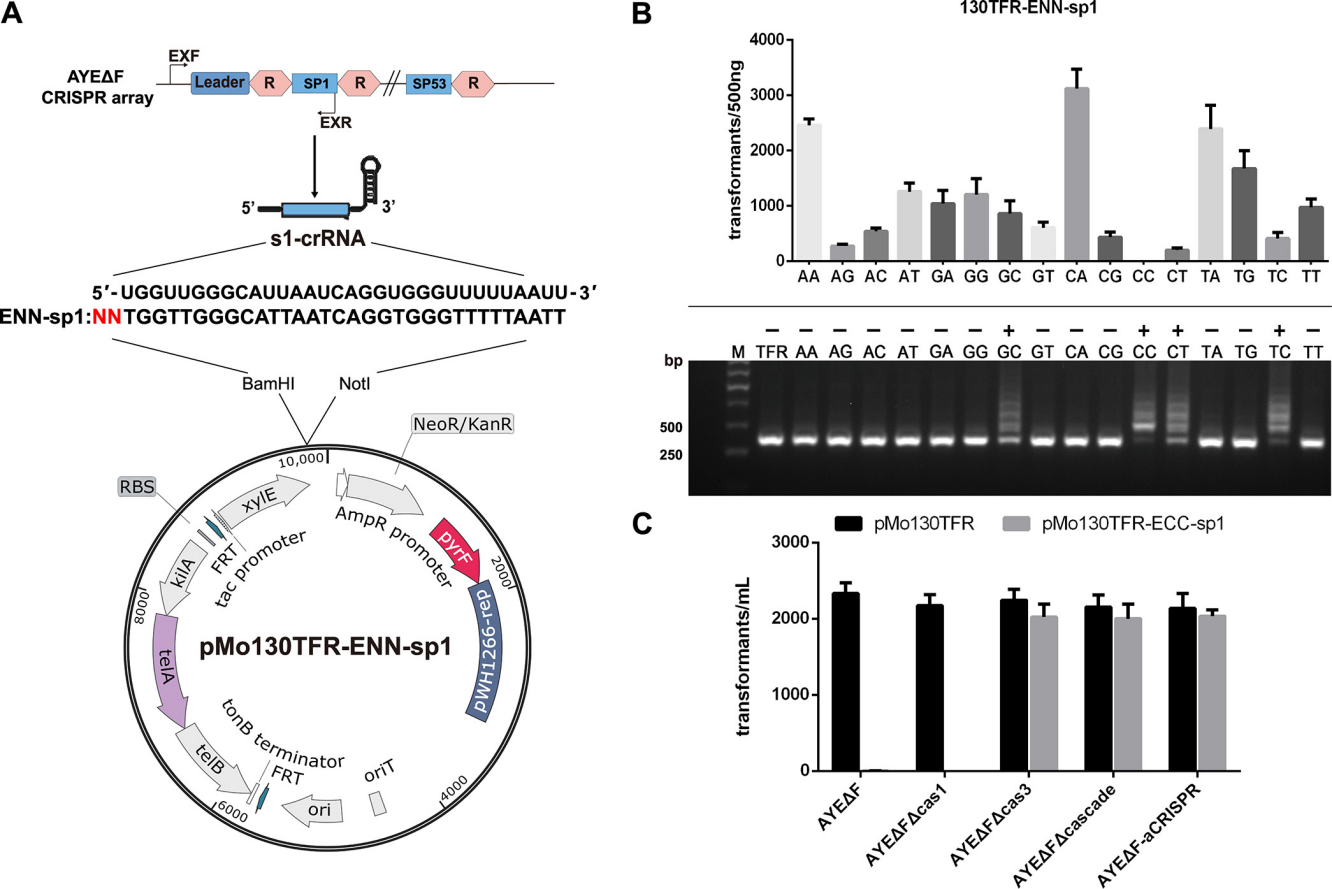
Use of the *pyrF*-based replicative plasmid for detecting the adaptation immunity of the I-F CRISPR-Cas system. (A) Schematic drawing of the strategy of constructing pMo130TFR-ENN-sp1 plasmids. The AYEΔF strains produced the s1 crRNA from the CRISPR, which can target the ENN-spacer 1 sequence in pMo130TFR-ENN-sp1. EXF/R primer pairs (black arrows) were designed against sequences that surround the CRISPR array for detecting expansion. (B) pMo130TFR-ENN-sp1 and pMo130TFR were transformed into AYEΔF in triplicate. Transformation efficiency was calculated as transformant counts. Moreover, expansion was detected by PCR amplification of the sequence flanking the CRISPR leader, and the subgenerations were used as the PCR template. The expanded bands (larger than the parental band) were produced by spacer insertion colonies (adaptation). −, pMo130TFR; M, marker. (C) The pMo130TFR-ECC-sp1 target plasmid and pMo130TFR (as the control) were each transformed into AYEΔFΔCas1, AYEΔFΔCas3, AYEΔFΔCascade, and AYEΔF-aCRISPR mutants in triplicate. Transformation efficiency was calculated as transformant counts.

In the I-F CRISPR-Cas system in A. baumannii, the precise cutting of the target gene sequence by CRISPR-Cas depends on specific recognition of the PAM, and the spacer acquisition also needs to recognize the PAM for priming adaptation. To explore the differences in the PAM recognition mechanism during the interference and priming adaptation phases of the I-F CRISPR immune process, the replicative plasmid pMo130TFR, which has high transformation efficiency, was utilized to generate the target plasmid pMo130TFR-ENN-sp1. A schematic drawing of the strategy used for investigation is shown in Fig. 4A. Sixteen pMo130TFR-ENN-sp1 target plasmids for AYEΔF were constructed, all of which carried a sequence that is fully matched by spacer 1 (protospacer 1) and had a preceding double nucleotide as the PAM sequence inserted in the 5′ end of spacer 1. These plasmids were transformed into uracil-auxotrophic AYEΔF cells under selection pressure. As shown in Fig. 4B, transformation assays demonstrated that the PAM-CC produced strongly enhanced interference effects in strain AYEΔF. Although each plasmid carries the fully matched spacer 1, the interference effects of some PAM mutations were not obviously reduced. This suggests that the I-F CRISPR-Cas system functioned well, and the PAM-CC sequence appeared to be necessary for interference. However, when these transformants were subincubated and adaptation was detected by PCR, the priming process was detected for target plasmids with PAM-GC, -CC, -CT, and -TC (Fig. 4B). These results demonstrated that interference recognized PAM-CC almost exclusively, while priming adaptation could tolerance more PAM mutations and was able to recognize PAM-GC, -CC, -CT, and -TC.

Based on the strongly enhanced interference effects of the replicative plasmid pMo130TFR-ECC-sp1, we utilized pMo130TFR-ECC-sp1 to verify the function of the above-described mutants with target gene deletions. pMo130TFR-ECC-sp1 and pMo130TFR (as the control) were transformed into A. baumannii AYEΔF, AYEΔFΔCas1, AYEΔFΔCas3, AYEΔFΔCascade, and AYEΔF-aCRISPR mutants. As shown in Fig. 4C, the target plasmid transformation efficiency for AYEΔFΔCas3, AYEΔFΔCascade, and AYEΔF-aCRISPR was similar to that for the control group, whereas the target plasmid transformation efficiency for AYEΔF and the Cas1 deletion mutant was obviously reduced. This suggests that almost all Cas proteins except Cas1 were involved in the interference process. The aCRISPR, with only the leader and repeat (without spacer 1), also could not detect the interference process, and therefore, priming is also necessary for adaptation in this I-F system.

### Exploring the vital DNA components of adaptive immunity for Acinetobacter baumannii I-F CRISPR-Cas.

**FIG 5:**
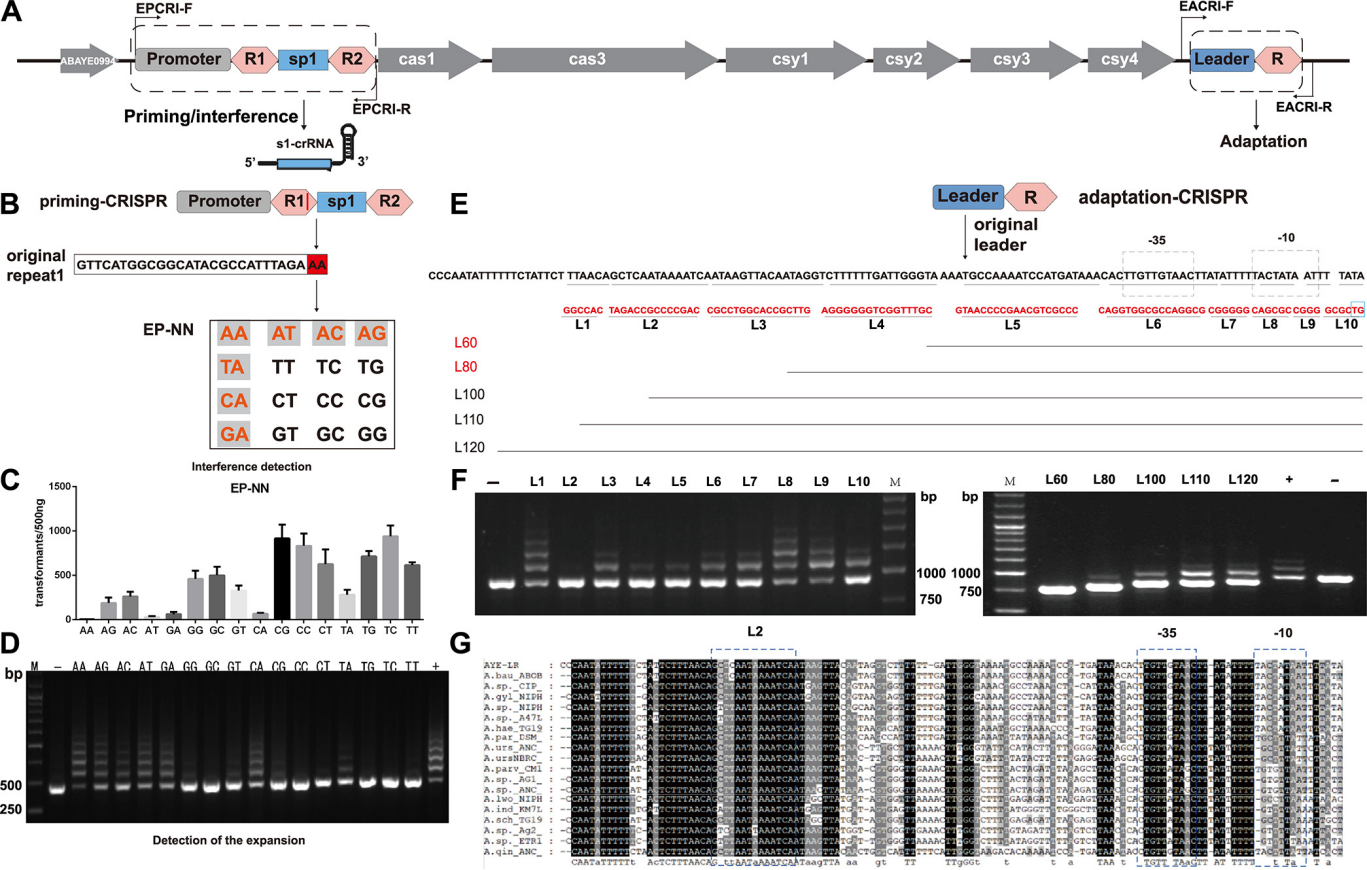
Assessment of the vital DNA components of adaptive immunity for Acinetobacter baumannii I-F CRISPR-Cas. (A) Construction of AYEΔF-PA. The pCRISPR carries a promoter and two repeat units flanking spacer 1. The aCRISPR carries the leader and a single repeat. EPCRI-F/R and EACRI-F/R primer pairs (black arrows) were designed against sequences that surround the pCRISPR and aCRISPR, respectively. (B) Modification of the double nucleotide at the end of the repeat 1 in priming CRISPR to construct EP-NN mutants. The 7 double nucleotides shown in red with a gray background are permissive for interference as well as for priming adaptation. (C) pMo130TFR-ECC-sp1 was transformed into the EP-NN mutants carrying double-nucleotide exchanges at the end of repeat 1, in triplicate. Transformation efficiency was calculated as transformants counts. (D) Detection of the EP-NN expansion by PCR amplifying the sequence flanking the CRISPR leader, and the subgenerations were used as the PCR template. The expanded bands (larger than the parental band) were produced by spacer insertion colonies (adaptation). −, pMo130TFR; +, AYEF transformants with pMo130TFR-ECC-sp1; M, marker. (E) Leader motifs of aCRISPR were modified to construct EA-L1, EA-L2, EA-L3, EA-L4, EA-L5 EA-L6, EA-L7, EA-L8, EA-L9, and EA-L10 mutants. The black dotted outlines indicate the −35 and −10 regions. Red sequences indicate mutations. The blue frame outlines the double-nucleotide sequence at the head of repeat 1 that was mutated. The aCRISPR leaders were truncated to construct EA-L60, EA-L80, EA-L100, EA-L110, and EA-L120 mutants. (F) Transformants with pMo130TFR-ECC-sp1 were inoculated into M95 medium by passage culture. Expansion was detected by PCR amplification of the sequence flanking the CRISPR leader, and the subgenerations were used as the PCR template. The expanded bands (larger than the parental band) were produced by spacer insertion colonies (adaptation). −, pMo130TFR; +, AYEF transformants with pMo130TFR-ECC-sp1; M, marker. (G) Conservative analysis of comparative the leader sequences of I-F CRISPR-Cas systems in Acinetobacter. The blue dashed line indicates the highly conserved sequence in the L2 region. Black dashed lines indicate the −35 and −10 regions.

In the native CRISPR system, a single CRISPR completes the processes of priming and spacer integration, which affects our research on the separate mechanisms of priming and spacer integration. Therefore, utilizing the *pyrF*/5-FOA genetic manipulation system, we established the AYEΔF-PA system with two CRISPR function systems for priming and spacer integration in AYEΔF (Fig. 5A). pCRISPR (containing a constitutive promoter and two repeat units flanking spacer 1 [pro-R1-sp1-R2]) was knocked into the genome to function as priming with plasmid pMo130TF-pCRISPR, while aCRISPR (containing the original leader and a single repeat) was modified to function as a new spacer integration site with plasmid pMo130TF-aCRISPR.

Previous data reported by Li et al. (39) demonstrated that the repeat end nucleotides preceding a spacer DNA distinguish target from nontarget. For systematic analysis of the importance and base preference of the type I-F repeat end for interference and priming, we mutated the double-nucleotide end of the repeat 1 in pMo130TF-pCRISPR and then constructed 16 pCRISPR mutants (EP-NN) based on AYEΔF-PA. As shown in Fig. 5B, transformation assays with pMo130TFR-ECC-sp1 demonstrated that the interference effects were enhanced when the end of repeat 1 was AA, AT, AC, AG, TA, CA, or GA and that the AA nucleotide exhibited the most efficiently enhanced interference effects. The results revealed that the role of double-nucleotide sequence mutations at the end of repeat 1 could be critical during CRISPR interference and have strong biases for A. Then, the transformants were inoculated into M95 medium. We used PCR and sequencing technology to detect the occurrence of the integration process (Fig. 5B). The priming effects were also enhanced when the end of repeat1 contained A, while expansion was not detected or not obviously detected in the PCR analysis if the end of repeat 1 lacked A. The results revealed that the role of double-nucleotide sequence mutations at the end of repeat 1 also could be important for the priming function, and especially biases for A. These similar biases showed that there is a close link between the priming process and the interference process, and the Cascade protein participates in these two processes, in which the identification of repeat 1 termini is important.

In the CRISPR immune system, the new spacer integration always occurred at the leader-proximal position during the adaptation process. Previous studies have shown that the leader motifs are very important for the identification of integration sites, but the nature of the recognition mechanism in type I-F CRISPR is not clear. Thus, we also truncated and mutated the leader sequence in pMo130TF-aCRISPR and then constructed aCRISPR mutants (EA-L) based on AYEΔF-PA, as shown in Fig. 5C. pMo130TFR-ECC-sp1 was also transformed into these mutants, and adaptations were detected by PCR and DNA sequencing. The EA-L2 mutation almost completely blocked the adaptation process. Obviously, L2 is rich in A bases, which showed that this sequence is critical for the adaptation process. We further constructed aCRISPR mutants containing various lengths of the leader sequence in aCRISPR, which were EA-L60, EA-L80, EA-L100, EA-L110, and EA-L120. The EA-L60 mutation completely and EA-L80 mutation almost completely blocked the adaptation process. EA-L60 and EA-L80 mutations both deleted the L2 region, which suggested that the L2 region of the repeat would be critical to the integration process in A. baumannii AYE. The sequence regions (L1 and L3 to L10) may affect the adaptation efficiency, but it will not completely block the integration process, indicating that this sequence is also important but not a key sequence.

## DISCUSSION

Acinetobacter baumannii is an important pathogenic microorganism due to the high mortality rates of nosocomial infection and high levels of extremely drug-resistant isolates. Convenient and quick genetic methods can help investigate the basic biology of A. baumannii infection and transmission and analyze the molecular mechanisms of gene function for these processes. In this study, we developed a widely applicable and efficient *pyrF*-based selection and counterselection system in A. baumannii for gene manipulation. Strains containing *pyrF* deletions as the uracil-auxotrophic hosts and corresponding *pyrF*-carrying suicide vectors constituted the gene knockout system for A. baumannii. In most cases, this *pyrF*/5-FOA genetic manipulation system was very effective and enabled us to obtain marker-free mutants in a very short period of time, while the *sacB*/sucrose system for plasmid eliminating from the genome was still difficult even after five subgenerations. As a result, the selection (uracil auxotroph) and counterselection (5-FOA) markers can be utilized for developing gene manipulation systems for broad and convenient application in model and clinical A. baumannii strains. Moreover, a series of vectors for gene manipulation were established and applied to validate the convenience and versatility. A limitation of this method is that if this gene manipulation system is used in clinical strains, the efficiency may be lower than that in model strains for various reasons, such as low transformation efficiency or biofilm formation. Recently, Wang et al. (49) reported the development of a highly efficient and scarless genome engineering platform in A. baumannii by coupling a Cas9-mediated genome cleavage system with the A. baumannii RecAb recombination system. The CRISPR-Cas9-based genome editing system and the *pyrF*/5-FOA system can both be utilized for developing gene editing systems for use in diverse A. baumannii strains. Moreover, these genetic manipulation methods are time-saving and efficient, and in particular, they can yield marker-free mutants. In addition, the deletion efficiencies of the pCasAb-pSGAb system of Wang et al. are higher than those of the *pyrF*/5-FOA system. On the other hand, the *pyrF*/5-FOA system needs to transfer only one plasmid into the recipient cell and the plasmid is removed easily, which makes it more convenient than the pCasAb-pSGAb system.

Based on the replicative *pyrF*-carrying plasmid, we explored the differences in the PAM recognition mechanism during the interference, priming, and acquisition phases of the I-F CRISPR immune process. Our results revealed that the PAM is also required to establish adaptive immunity in the 5′ end of identical protospacer sequences in the invading DNA and suggest that A. baumannii AYE interference almost exclusively recognizes the PAM-CC sequence, while priming adaptation could tolerance more PAM mutations and recognized PAM-GC, -CC, -CT, and -TC. Three of the four PAM sequences were generated by CC through a single point mutation, suggesting that these PAM mutations in the escape interference process drove the evolution of PAM selectivity in the adaptation process. The greater PAM selectivity during the adaptive immunity process explains the tolerance of PAM mutations in a target. Subsequently, based on the separate mechanisms of interference and/or primed adaptation, we mutated the double-nucleotide sequence at the end of repeat 1, which is transcribed as double nucleotides of the 5′ handle of crRNA. We observed the preference for A at the −1 and −2 positions in the interference and priming adaptation processes, which corresponds to native repeat motifs (the AA nucleotides occur at the end of repeat 1 of native I-F CRISPR-Cas in A. baumannii), which suggested that priming adaptation and interference can tolerate nucleotide variations within the crRNA molecule.

These data exclude the possibility that the type I-F system makes use of a base pairing between the crRNA spacer and the protospacer to inhibit self-targeting. The priming adaptation and interference processes both require crRNA to target the protospacer based on base pairing and require the PAM verification mechanism to avoid targeting spacer DNA. These processes possess different tolerances for mutations in the PAM and the 5′ handle of crRNA at positions −1 and −2.

We hope that providing the selection of PAM will establish a basis for furthering the understanding of the functional target and nontarget recognition mechanisms at the CRISPR site in A. baumannii. The Pseudomonas aeruginosa type I-F system targets foreign DNA through complex mechanisms, involving protein-mediated interaction with DNA and crRNA-guided interaction with cDNA and has been proposed as the model of the Csy complex target search process (50). Based on that and a model for a Cascade-mediated recognition mechanism proposed in a previous study (39), we envisioned a discrimination mechanism for CRISPR interference and priming adaptation. We speculated that the Cascade (CRISPR-associated complex for antiviral defense) complex may utilize its protein subunit(s) to distinguish target from nontarget by PAM scanning, and the interference and priming adaptation can avoid CRISPR DNA becoming a potential target, because the PAM is absent from repeat sequences in the host CRISPR locus and is present only next to complementary protospacer targets in foreign DNA. The binding affinities for Cascade complex with crRNA and the protospacer DNA with alternate PAMs results in the PAM and 5′ handle of CRISPR repeat having different nucleotide tolerances for the CRISPR interference and adaptation-priming processes, and we speculate that the double nucleotide at the end of repeat 1 (−1 and −2 positions in crRNA) may be responsible for regulating the proper anchoring between the 5′ handle repeat of crRNA and Cascade complex. After a PAM that allowed interference (PAM-CC) or adaptation (PAM-GC, -CC, -CT, and -TC) to occur was detected, the Cascade complex with crRNA was used to further examine whether the target sequence exactly matched the crRNA spacer sequence. When the protospacer exactly matches the crRNA spacer DNA, interference or adaptation will occur. The reasons for PAM selectivity and A affinity require a more sophisticated model and further experiments to explain.

The leader motifs are also important for the identification of integration sites. To analyze the key recognition and acquisition mechanism in type I-F CRISPR, for gaining experimental insight into the comparative sequence analysis of I-F leader motifs in Acinetobacter, we mutated leader sequences and lengths that regulate CRISPR adaptation. Our results showed that the L2 region is rich in A bases and is critical for the adaptation process to acquire new spacers. This region was determined to be conserved by comparative analysis of the leader sequences of I-F CRISPR-Cas systems in Acinetobacter. Therefore, this observation reflected a specific conservative leader sequence pattern and regulation in the I-F CRISPR. We observed that a leader shortened to 60 bp does not support detectable acquisition. The high rate of spacer acquisition was obtained with at least 100 bp of the full leader sequence. The type I-E and I-F systems have been shown to rely on a DNA-bending protein called integration host factor (IHF). In type I CRISPR systems, identified leader DNA sequences are specifically recognized by the IHF protein to facilitate leader-proximal spacer integration (51). The mechanisms of CRISPR adaptation are diverse, but all systems that rely on IHF are expected to be phase dependent (51, 52). We hypothesized that the L2 region DNA in the leader may bind IHF protein sites, which creates a stabilized structure with the Cas1-2 integrase complex bound to the first repeat of the CRISPR locus and determines the appropriate integration site at the leader-repeat junction (44–46, 51, 53), but specific structural analysis is required to further investigate this. Moreover, the phase of leader motifs, rather than their distance from the leader-repeat junction, may be critical for efficient adaption in IHF-dependent systems.

Our goal was to further clarify the mechanism of site-specific integration and provide a theoretical basis for limiting the transmission of virulence and drug resistance genes of Acinetobacter baumannii by the *pyrF*/5-FOA genetic manipulation system. This *pyrF*-based system was more suitable than antibiotics to modify the DNA motifs for exploring the vital DNA components of adaptive immunity in Acinetobacter baumannii I-F CRISPR-Cas, and it provides us with a feasible gene tool for gene manipulation in the laboratory. In conclusion, the *pyrF*-based system is an efficient and convenient system for broad use in most A. baumannii strains to study drug resistance, virulence, and other gene functions.

## MATERIALS AND METHODS

### Bacterial strains, plasmids, and culture conditions.

The strains, plasmids, and primers used in this study are listed in Table S1. E. coli strains were grown at 37°C in Luria-Bertani (LB) medium, supplemented with the appropriate agent. A. baumannii strains and mutants were grown at 37°C in LB medium, supplemented as needed with potassium tellurite (Tel; 30 mg/L), kanamycin (Km; 50 mg/L), uracil (U; 50 mg/L), 5-fluoroorotic acid (5-FOA; 50 mg/L), and agar (15 g/L). For pyrimidine-free medium, a synthetic medium (M95) was used, which comprised 0.6% Na_2_HPO_4_, 0.3% KH_2_PO_4_, 0.1%NH4Cl, 1%NaCl, 1 × 10^−5^% thiamine-HCl, 0.2% glucose, 1 mM MgSO_4_, and 0.5% Bacto Casamino Acids (pH 7.0).

### Bioinformatics methods and molecular biology.

The gene sequences analyzed in this study were obtained from NCBI (https://www.ncbi.nlm.nih.gov/); amino acid and nucleotide sequences in A. baumannii were analyzed through BLASTP and BLAST. All cloning steps were performed in E. coli DH5α. The primers used for vector construction and PCR detection are listed in Table S2. PCR was performed according to the manufacturer′s instructions by using Phanta Max master mix (Vazyme) for cloning and 2× *Taq* master mix (Vazyme) for detection. Restriction enzymes used for cloning were from New England BioLabs (Ipswich, MA). Plasmids were all constructed through homologous recombination technology using a ClonExpress II one-step cloning kit (Vazyme) or T4 DNA ligase (Vazyme).

### Plasmid construction.

**(i) Construction of the plasmid for *pyrF* gene deletion.** The pMo130Tel^R^-ΔF vector was designed to delete the entire open reading frame of the *pyrF* gene in A. baumannii. The upstream and downstream 500-bp regions flanking *pyrF* in A. baumannii AYE were PCR amplified with the primers Δ*pyrF*-UF/UR and Δ*pyrF*-DF/DR and cloned into pMo130Tel^R^ (BamHI and PstI digested) to generate pMo130Tel^R^-ΔF.

**(ii) Suicide plasmids with *pyrF* as a selectable and counterselectable marker.** The *pyrF* gene (along with its 200-bp upstream promoter sequence) was PCR amplified with the primer pair *pyrF*-F/R. Subsequently, this fragment was inserted into the EcoRI/KpnI sites of pMo130Tel^R^ to replace the *sacB* gene for generating pMo130TF. Similarly, this *pyrF* gene was inserted into the EcoRI/KpnI sites of pMo130 to replace the *sacB* gene for generating pMo130F. The pMo130TF and pMo130F vectors were suicide plasmids for homologous recombination, which used the *pyrF* gene as the selectable and counterselectable marker.

**(iii) Autonomous replication plasmids with *pyrF* as a selectable marker.** pMo130TFR and pMo130FR were autonomous replication plasmids, which did not need to integrate into the genome and could use the *pyrF* gene as a selection marker. The *rep* gene of pWH1266 was PCR amplified and cloned into pMo130TF (EcoRI digested) and pMo130F (EcoRI digested) to generate pMo130TFR and pMo130FR with the primer pair Rep-F/R.

**(iv) Reporter and induced plasmids with *pyrF* as a selectable marker.** Based on pMo130TFR, we constructed the GFP reporter plasmid pMo130TFRG-P_tac_ and the IPTG-induced expression plasmid pMo130TFRI. To construct pMo130TFRG-P_tac_, the *tac* promoter region and GFP-mut3 with a strong bacterial RBS gene, AAAGAGGAGAAA (54), were amplified with the primers Tac-GFP-F1/F2/F3/R and inserted into the BamHI/PstI sites of pMo130TFR. The *lacI* gene and lac operator were inserted into pMo130TFRG-P_tac_ (BamHI and SphI) to construct pMo130TFRI with the primers LacI-F/R.

**(v) Plasmids for gene manipulation.** For gene deletion, the upstream and downstream 500-bp flanking regions of Cas1, Cas3, Cascade, and a large part of the CRISPR genes except the leader and a single repeat structure (aCRISPR) were PCR amplified and cloned into pMo130TF (BamHI and PstI digested) or pMo130Tel^R^ (BamHI and PstI digested) to generate pMo130TF-ΔCas1, pMo130TF-ΔCas3, pMo130TF-ΔCascade, pMo130TF-aCRISPR, pMo130Tel^R^-ΔCas1, pMo130Tel^R^-ΔCas3, pMo130Tel^R^-ΔCascade, and pMo130Tel^R^-aCRISPR with the primer pairs ΔCas1-UF/UR, ΔCas1-DF/DR, ΔCas3-UF/UR, ΔCas3-DF/DR, ΔCascade-UF/UR, ΔCascade-DF/DR, ΔCRISPR-UF/UR, and ΔCRISPR-DF/DR, respectively.

**(vi) Plasmids for CRISPR interference and adaptation detection.** The pMo130TFR-ENN-sp1 plasmids are target plasmids for detecting CRISPR interference and adaptation. To construct pMo130TFR-ENN-sp1 target plasmids, nucleotide substitutions were performed by PCR mutagenesis. The PAM 5′-NN and spacer 1 of the AYE CRISPR-Cas system were PCR amplified with primer ENN-sp1-F/R and cloned into pMo130TFR (NotI and BamHI digested). The possible base composition of the PAM sequence was AA, AT, AC, AG, TA, TT, TC, TG, CA, CT, CC, CG, GA, GT, GC, or GG, yielding 16 different plasmids, each named in the format pMo130TFR-ENN-sp1, where NN represents the PAM sequence and 1 represents protospacer 1. E. coli S17-1 was transformed with the pMo130TFR-ENN-sp1 plasmids and pMo130TFR (as the control) and subsequently served as the donor strain in mating with AYEΔF.

### Transformation.

Electrocompetent A. baumannii wild-type and mutant cells were prepared by inoculating a fresh colony and grown at 37°C overnight with 220 rpm agitation. One-milliliter cultures in the late logarithmic phase were centrifuged at 10,000 rpm and 4°C for 2 min. The harvested cells were washed three times with 10% cold glycerol and then resuspended in 100 μL of 10% cold glycerin for transformation.

For electrical transformation, 500 ng of the autonomously replicating plasmids pMo130TFR and their derivative plasmids is needed for mixing with 100 μL of competent cells, while 10 μg of the suicide plasmids and their derivative plasmids is needed. The mixed cells were incubated on ice for 10 min and then transferred to a 1-mm cuvette at a voltage of 1,800 V, a capacitance of 25 μF, and a resistance of 200 Ω. After electroporation, the competent cells were transferred to a 1.5-mL microfuge tube with 500 μL LB liquid medium added and cultured at 200 rpm and 37°C for 45 min. The cells were plated on Tel-containing LB plates or M95 plates and cultured overnight at 37°C.

### Isolation and characterization of mutants with a gene knockout system.

After electrical transformation, the cells were plated on Tel-containing LB plates or M95 plates to select the colonies that contained *pyrF*-carrying plasmids either integrated into the genome or autonomously replicated. For gene deletion or mutation by the *pyrF*/5-FOA system, excision of the plasmids integrated in the genome to screen the mutant colonies was performed by propagating in LB medium containing uracil (50 mg/L) and 5-FOA (50 mg/L) as a counterselection agent. For the *sacB*/sucrose system, we used sucrose (10%) as the counterselection agent to manipulate genes as previously described (16). The colonies were picked and streaked on replica plates (the same colony was streaked on LB plates and continuously streaked on LB plates supplemented with 30 mg/L Tel to confirm that the *pyrF* genes or target genes were eliminated from the genome) and then PCR amplified to screen the colonies with the correct size band and DNA sequencing.

### Measuring growth curves and susceptibility to 5-FOA.

The wild-type strains and mutants of A. baumannii were streaked on both LB plates and LB plates with 5-FOA (50 mg/L) and cultured overnight to test susceptibility to 5-FOA. For the uracil-prototrophic strains of A. baumannii, we measured the growth curve by liquid culture in both LB and LB with uracil (50 mg/L) and also streaking on M95 plates and M95 plates with uracil (50 mg/L).

### Detection of the fluorescence/OD_600_ ratio.

The gene for the GFP-mut3 protein was linked to the *tac* promoter using the overlap extension PCR strategy. The *pyrF*-carrying plasmids were transformed into AYEΔF. For each transformation assay, three individual colonies were selected and cultured to the late exponential phase with or without 0.5 mM IPTG, and their OD_600_ and fluorescence were simultaneously determined using the Synergy H4 hybrid multimode microplate reader (BioTek, VT, USA). The fluorescence/OD_600_ ratio was calculated for each of the three individual samples, and averages and standard deviations were calculated.

### Construction of CRISPR mutants.

We established a system with two CRISPR systems, priming CRISPR (pCRISPR) and adaptation CRISPR (aCRISPR) structures, the former for detecting the priming and interference process and the latter for containing the complete CRISPR leader to incorporate new spacers. An integrative plasmid, pMo130TF-pCRISPR, was first constructed, and it was transformed into AYEΔF-aCRISPR (AYEΔF with a large part of CRISPR deleted except the original leader and a single repeat structure) to yield AYEΔF-Pa with pCRISPR mutants and the original aCRISPR. The upstream homologous arm fragment (chromosomal sequences immediately upstream of Cas1) was amplified with the primers pCRISPR-FPF-SphI and pCRISPR-FPR-BamHI, and the downstream homologous arm fragment (chromosomal sequences immediately downstream of fragments of the ABAYE0994 gene) was amplified with primers pCRISPR-PPF-PstI/pCRISPR-PPR-NotI, while the promoter and two repeat units flanking spacer 1 (a variant pro-R1-sp1-R2 structure named priming-CRISPR) were amplified with the primers pCRISPR-F1-BamHI and pCRISPR-R1-PstI. The fragments were joined with suicide plasmid pMo130TF (NotI and SphI digested) to generate PCRI (Table S1) with T4 DNA ligase (Vazyme). Subsequently, the aCRISPR was designed to transform into AYEΔF-Pa to gain the AYEΔF-PA with a-CRISPR mutants. The chromosomal sequences upstream (aCRISPR-FPF-SphI/aCRISPR-FPR-BamHI) and downstream (aCRISPR-PPF-PstI/aCRISPR-PPR-NotI) of the AYE wild-type CRISPR, along with the leader and a single repeat (a variant leader R1 structure named adaptation CRISPR) with primers aCRISPR-F1-BamHI and aCRISPR-R1-PstI, were cloned into the suicide plasmid pMo130TF (NotI and SphI digested) to generate pMo130TF-aCRISPR by the same method. pMo130TF mutated double-nucleotide exchanges at the end of repeat 1 to yield the EP-NN mutants, which were transformed into cells for detecting the priming and interference process. pMo130TF-aCRISPR mutated the original leader motifs to yield the EA-L1~L10 and EA-L60~L120 mutants. All mutated CRISPRs were subjected to PCR mutagenesis and validated by PCR analysis and DNA sequencing with p130-F/R primers and were screened through the gene knockout strategy as described above.

### Detection of CRISPR interference.

The target plasmids were transformed into A. baumannii or its derivative strain by electrical transformation. For each target plasmid, at least three independent replicates were performed. For CRISPR interference detection, we counted the transformant colonies and conducted a comparative analysis.

### Detection of spacer integration.

To monitor spacer acquisition from the plasmid DNA, the transformants of each target plasmid were inoculated into M95 medium by passage culture, and the subgenerations were used as the colony PCR template (using the primer pair EXF/R or EACRI-F/R). PCR amplification of the sequence flanking the leader can detect the expansion. The PCR program consisted of the following steps: (i) 95°C for 3 min; (ii) 30 cycles of 95°C for 30 s, 55°C for 30 s, and 72°C for 30 s; (iii) 72°C for 10 min. The PCR products were separated on a 2% agarose gel. The expanded bands (larger than the parental band) were produced by spacer insertion colonies (adaptation).
