# Supplementary material for: A pyrF-Based Efficient Genetic Manipulation Platform in Acinetobacter baumannii To Explore the Vital DNA Components of Adaptive Immunity for I-F CRISPR-Cas
Source: Microbiol Spectr. 2022 Sep 1;10(5):e01957-22. doi: 10.1128/spectrum.01957-22 (PMC9602844; doi:10.1128/spectrum.01957-22)
Supplement: Supplemental file 1 — Fig. S1 to S4 and Tables S1 and S2. Download spectrum.01957-22-s0001.pdf, PDF file, 2.2 MB [file spectrum.01957-22-s0001.pdf]

A *pyrF*-based efficient genetic manipulation platform in *Acinetobacter baumannii* to explore the vital DNA components of adaptive immunity for I-F CRISPR-Cas

Supplementary document

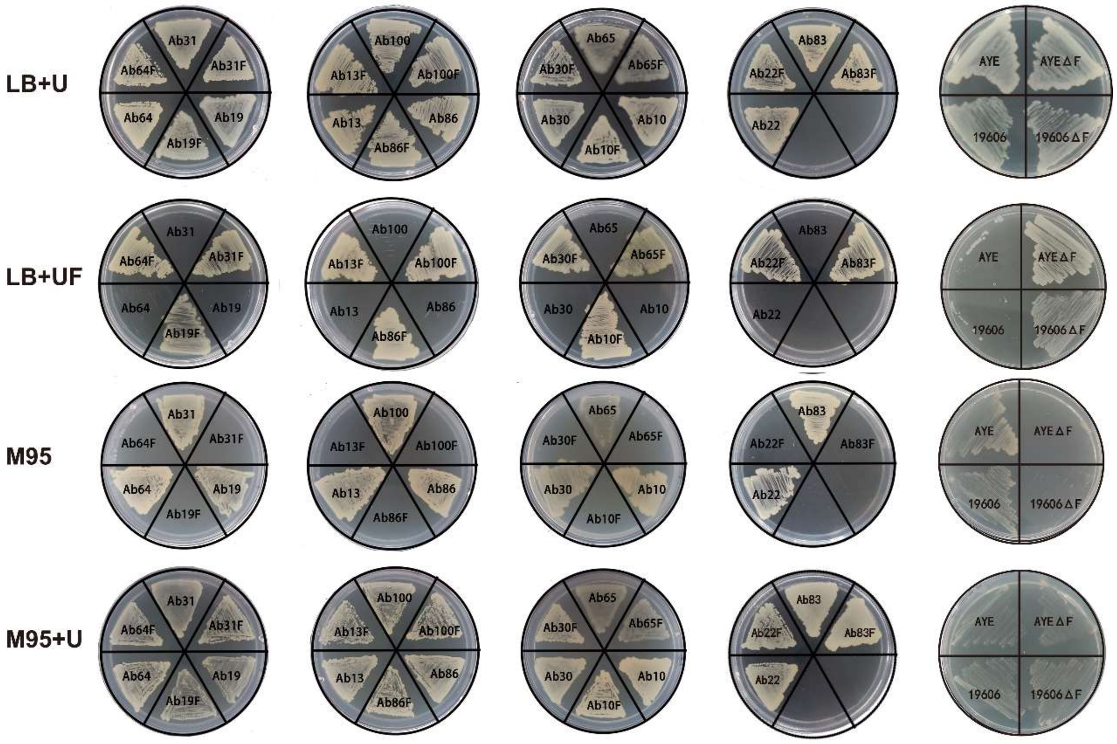

FIG S1. The uracil prototrophic and the sensibility to 5-FOA for two model and 11 clinical strains *A. baumannii* and their *pyrF*-deleted mutants. LB+UF plates were detected the sensibility to 5-FOA, with LB+U plates as the control. M95 plates were detected the uracil auxotrophic, with M95+U plates as the control. Wild type strains: AYE, 19606, Ab64, Ab19, Ab31, Ab13, Ab100, Ab86, Ab65, Ab30, Ab10, Ab22, and Ab83; *pyrF*-deleted mutants: AYEΔF, 19606ΔF, Ab64ΔF, Ab19ΔF, Ab31ΔF, Ab13ΔF, Ab100ΔF, Ab86ΔF, Ab65ΔF, Ab30ΔF, Ab10ΔF, Ab22ΔF, and Ab83ΔF.

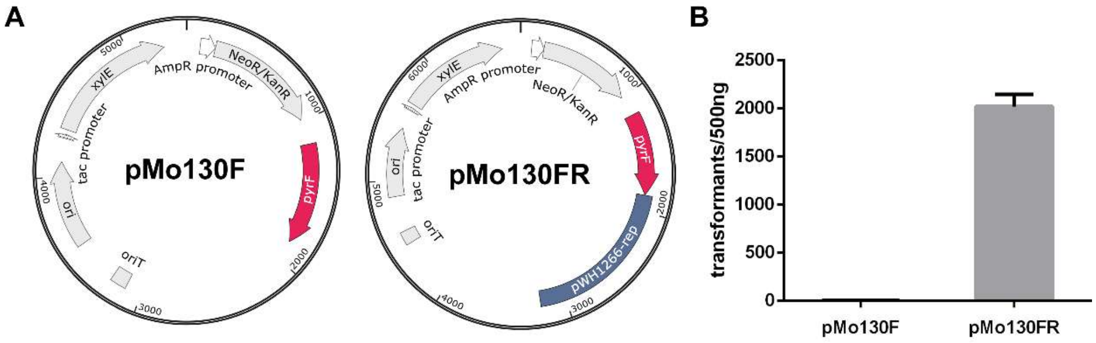

FIG S2. A. The *pyrF* gene (along with its 200bp-upstream promoter sequence) was introduced into pMo130 to replace the *sacB* gene to generate the pMo130F. The rep gene of pWH1266 was introduced into pMo130F to generate the pMo130FR, respectively. B. The pMo130FR and pMo130F were respectively transformed into AYEΔF in triplicate. The transformation efficiency was shown.

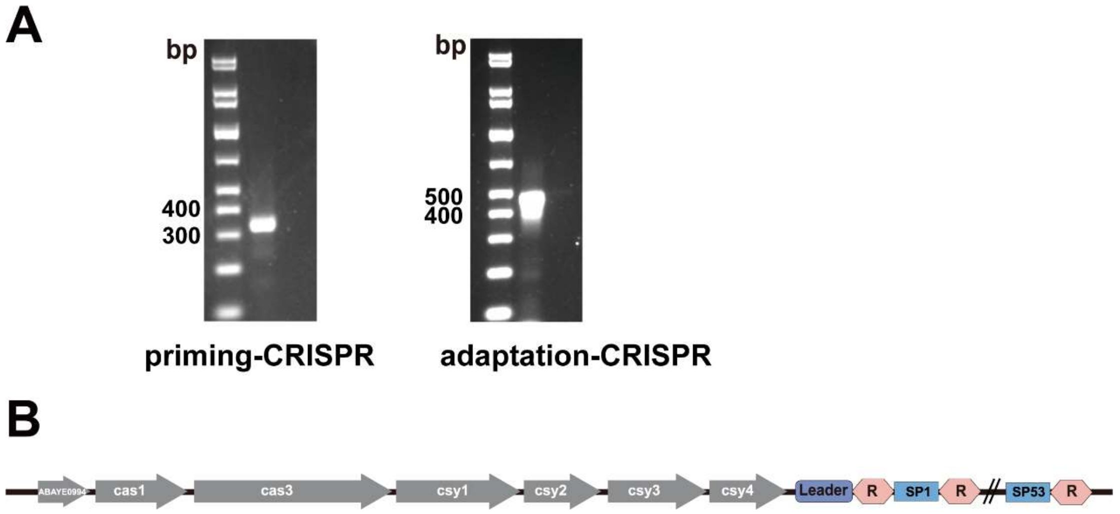

FIG S3. A. The pMo130TF-pCRISPR and pMo130TF-aCRISPR plasmids were constructed by a PCR validation. The expected length was 341 bp for the pCRISPR and 435bp for the aCRISPR. B. the CRISPR structure of the *A. baumannii* wild strains.

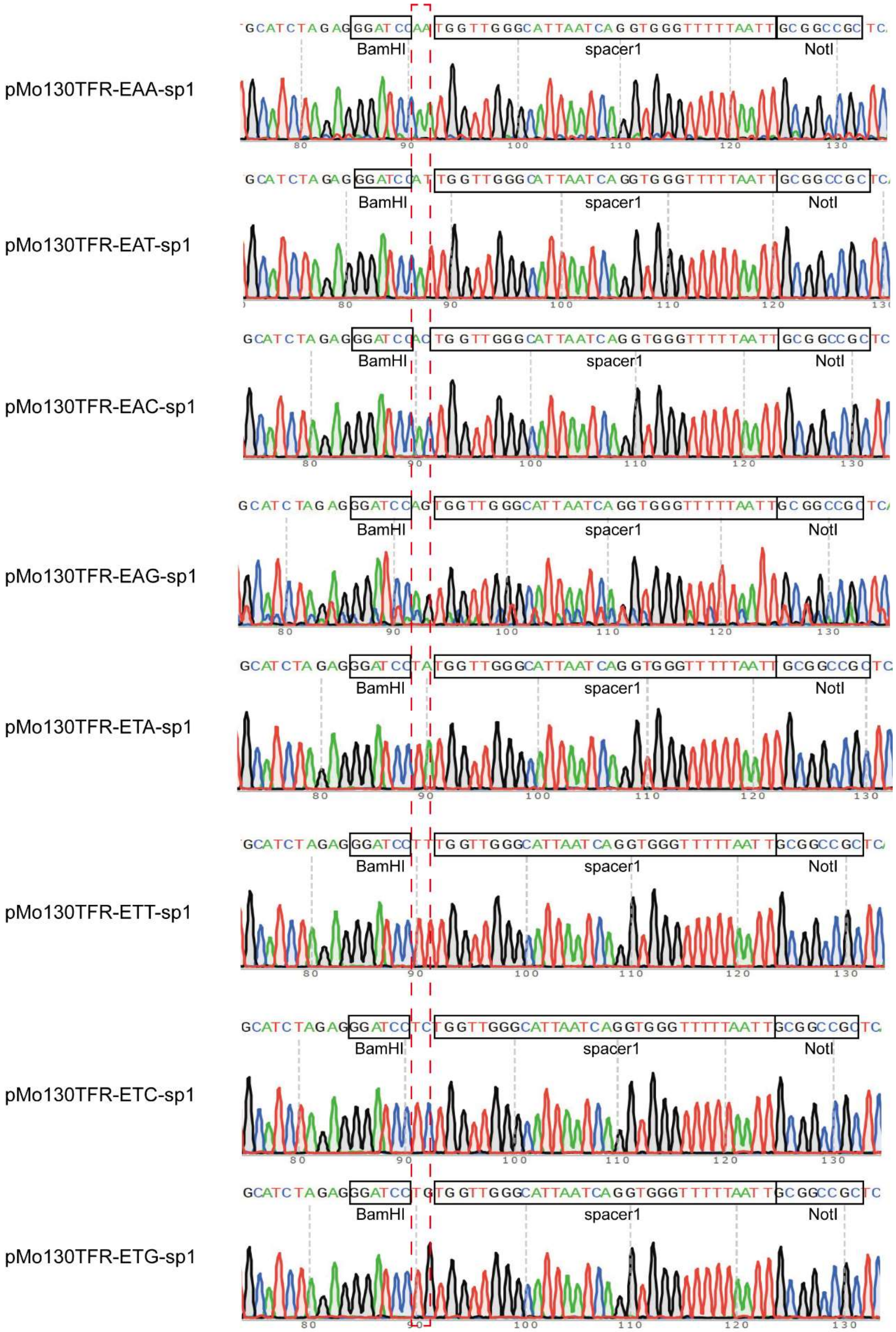
BamHI
spacer1
NotI
pMo130TFR-EAA-sp1
pMo130TFR-EAT-sp1
pMo130TFR-EAC-sp1
pMo130TFR-EAG-sp1
pMo130TFR-ETA-sp1
pMo130TFR-ETT-sp1
pMo130TFR-ETC-sp1
pMo130TFR-ETG-sp1

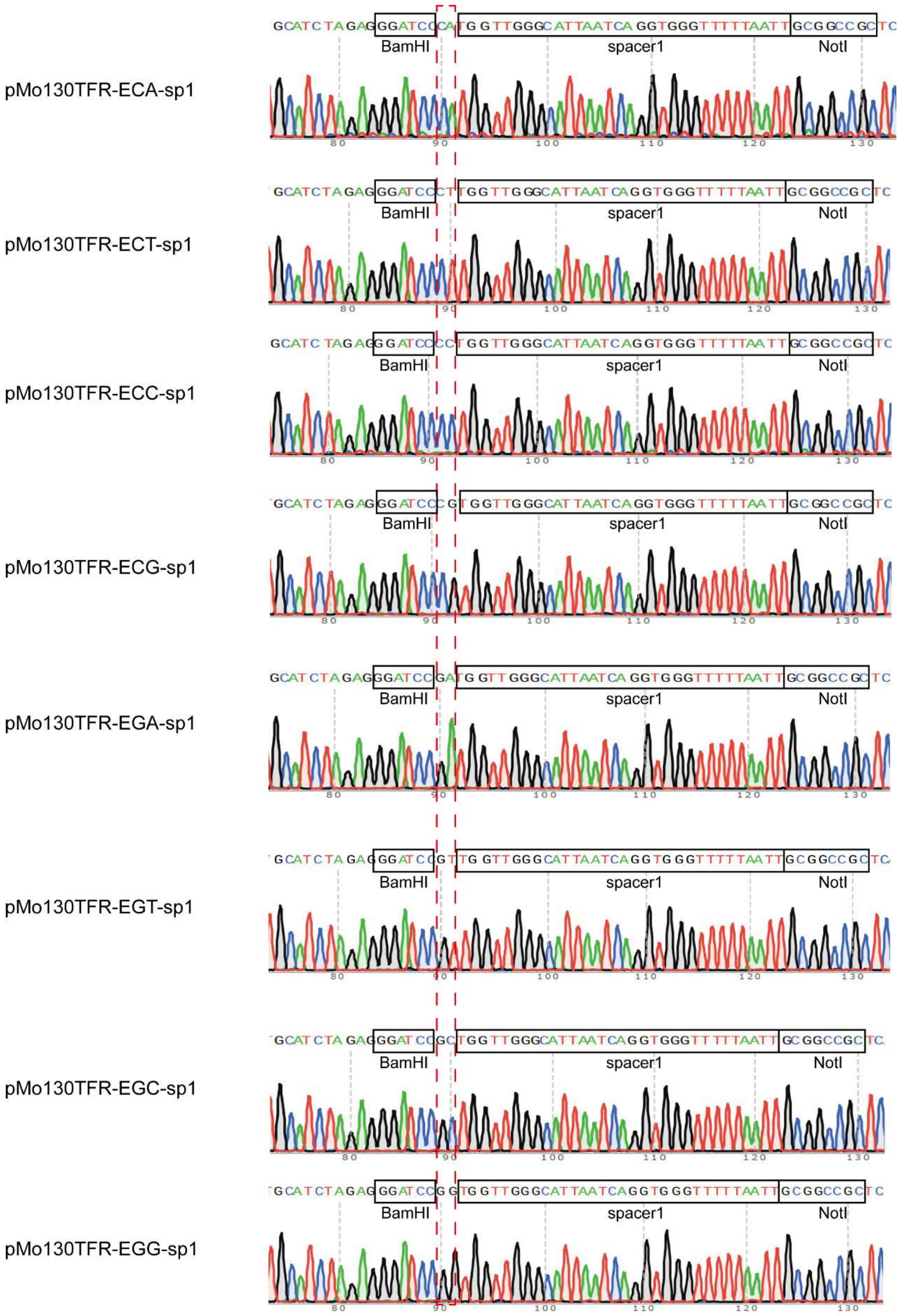
pMo130TFR-ECA-sp1
BamHI
spacer1
NotI
pMo130TFR-ECT-sp1
BamHI
spacer1
NotI
pMo130TFR-ECC-sp1
BamHI
spacer1
NotI
pMo130TFR-ECG-sp1
BamHI
spacer1
NotI
pMo130TFR-EGA-sp1
BamHI
spacer1
NotI
pMo130TFR-EGT-sp1
BamHI
spacer1
NotI
pMo130TFR-EGC-sp1
BamHI
spacer1
NotI
pMo130TFR-EGG-sp1
BamHI
spacer1
NotI

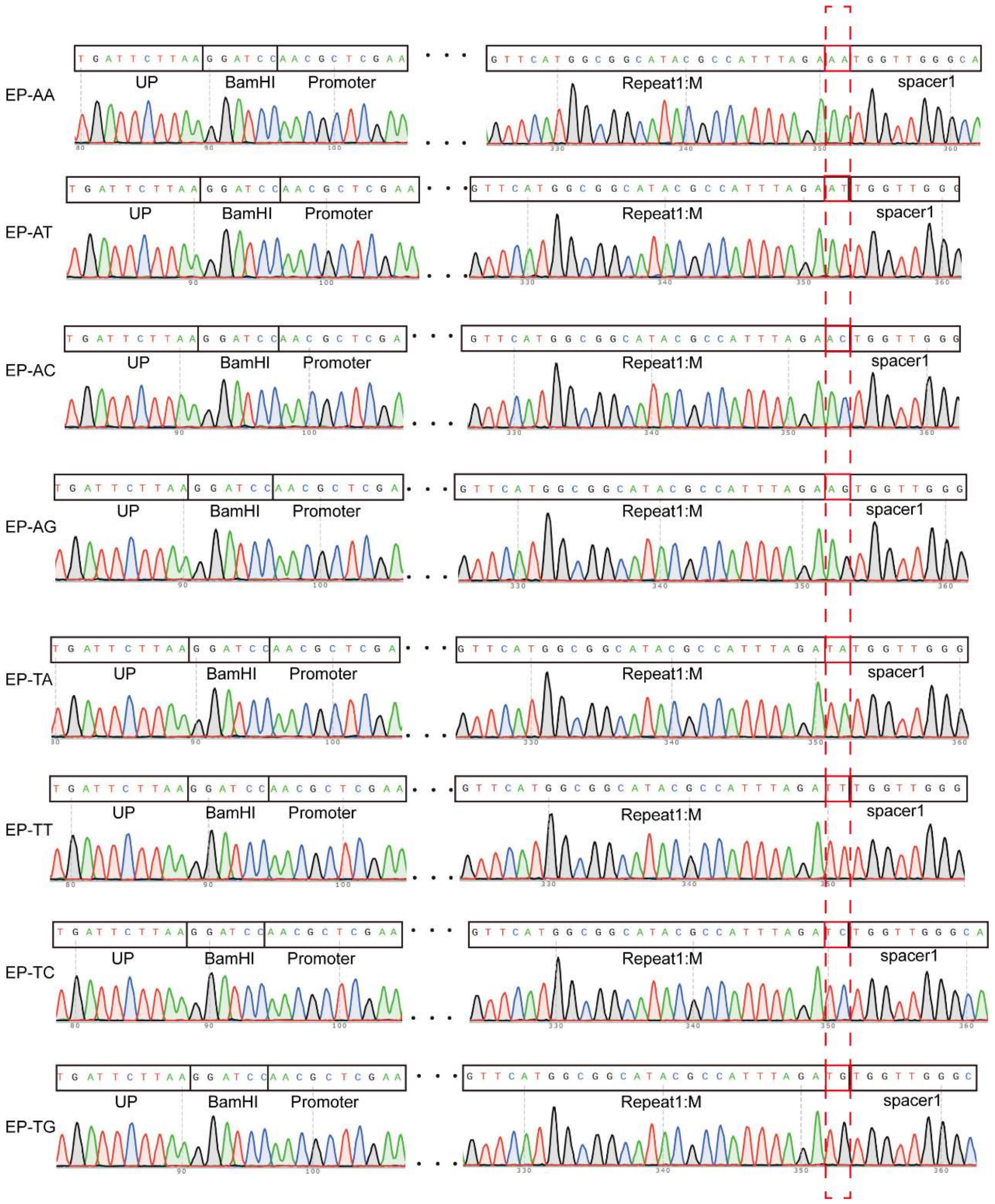
EP-AA
EP-AT
EP-AC
EP-AG
EP-TA
EP-TT
EP-TC
EP-TG
UP
BamHI
Promoter
Repeat1:M
spacer1

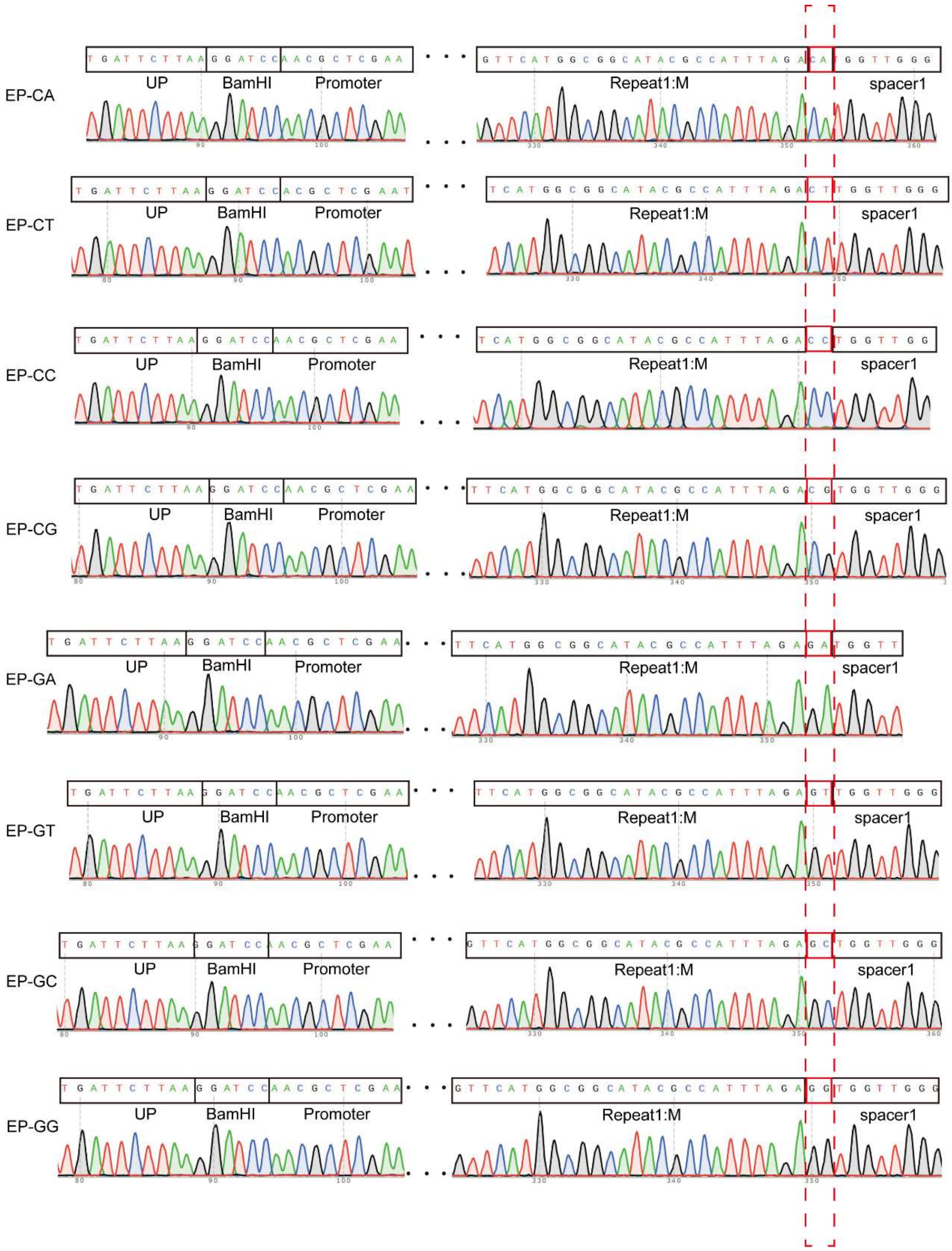

EP-CA
EP-CT
EP-CC
EP-CG
EP-GA
EP-GT
EP-GC
EP-GG
UP
BamHI
Promoter
Repeat1:M
spacer1

42

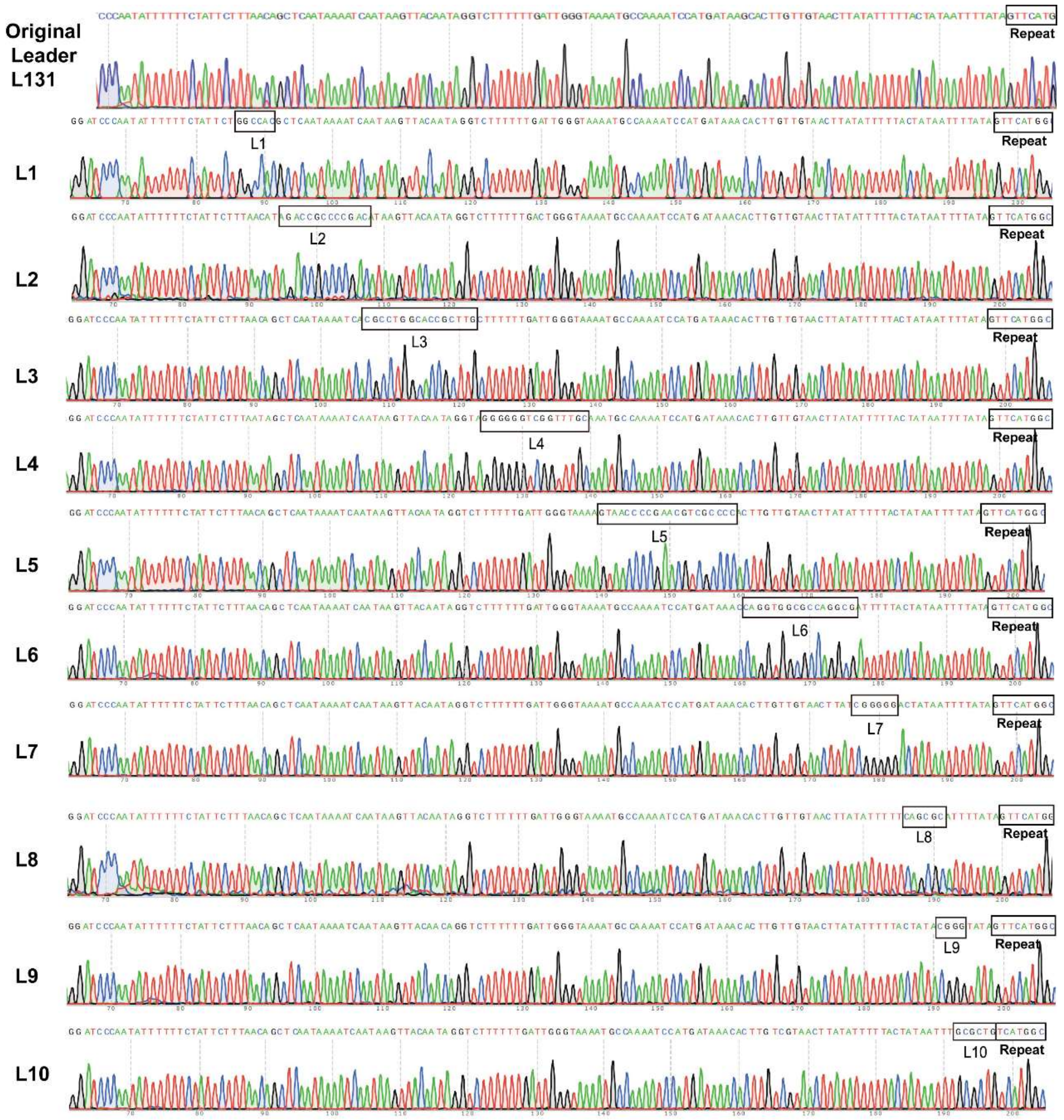
Original Leader L131
Repeat
L1
L2
L3
L4
L5
L6
L7
L8
L9
L10

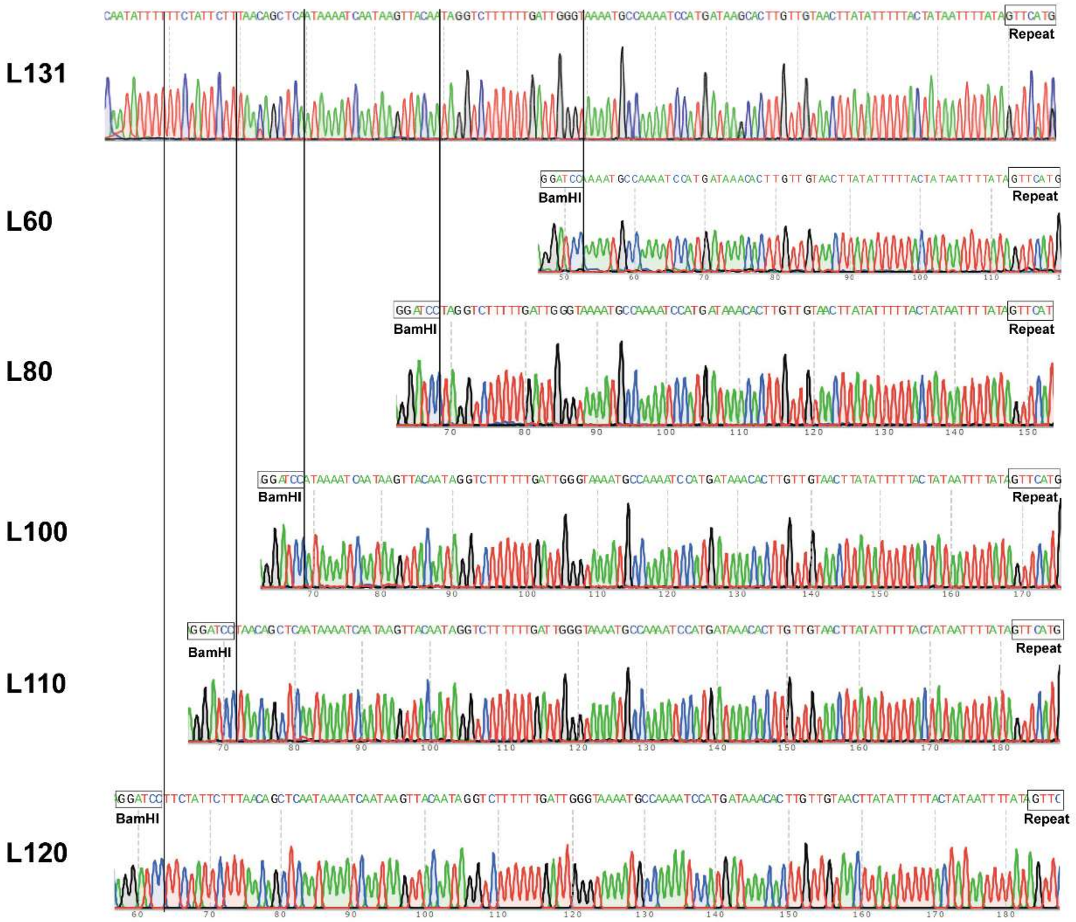

FIG S4. Sanger sequencing of pMo130TFR-ENN-sp1, EP-NN, EA-L1 to L10, and EA-L60 to L120 mutants

TABLE S1 The strains and plasmids used in this study.

Strains or plasmids	Relevant characteristics	Source or reference
A.*baumannii* AYE	Model strain of A.*baumannii*	ATCC
A.*baumannii* AYEΔF	Δ*pyrF* mutant of A.*baumannii* AYE	This study
A.*baumannii* ATCC19606	Model strain of A.*baumannii*	ATCC
A.*baumannii* 19606ΔF	Δ*pyrF* mutant of A.*baumannii* 19606	This study
E. coli DH5α strains		Invitogen
S17-1	Donor strain for suicide plasmid transfer	This study
AYEΔFΔCas1	ΔCas1 mutant of AYEΔF	This study
AYEΔFΔCas3	ΔCas3 mutant of AYEΔF	This study
AYEΔFΔCascade	ΔCascade mutant of AYEΔF	This study
AYEΔF-aCRISPR	AYEΔFΔCRISPR with original adaptation CRISPR mutants	This study
AYEΔF-PA	AYEΔFΔCRISPR introduced priming CRISPR and adaptation CRISPR mutations	This study
EP-AT	Modified AYEΔF-PA to mutate double-nucleotide at the end of the repeat1 AA to AT	This study
EP-AC	Modified AYEΔF-PA to mutate double-nucleotide at the end of the repeat1 AA to AC	This study
EP-AG	Modified AYEΔF-PA to mutate double-nucleotide at the end of the repeat1 AA to AG	This study

EP-TA	Modified AYEΔF-PA to mutate double-nucleotide at the end of the repeat1 AA to TA	This study
EP-TT	Modified AYEΔF-PA to mutate double-nucleotide at the end of the repeat1 AA to TT	This study
EP-TC	Modified AYEΔF-PA to mutate double-nucleotide at the end of the repeat1 AA to TC	This study
EP-TG	Modified AYEΔF-PA to mutate double-nucleotide at the end of the repeat1 AA to TG	This study
EP-CA	Modified AYEΔF-PA to mutate double-nucleotide at the end of the repeat1 AA to CA	This study
EP-CT	Modified AYEΔF-PA to mutate double-nucleotide at the end of the repeat1 AA to CT	This study
EP-CC	Modified AYEΔF-PA to mutate double-nucleotide at the end of the repeat1 AA to CC	This study
EP-CG	Modified AYEΔF-PA to mutate double-nucleotide at the end of the repeat1 AA to CG	This study
EP-GA	Modified AYEΔF-PA to mutate double-nucleotide at the end of the repeat1 AA to GA	This study
EP-GT	Modified AYEΔF-PA to mutate double-nucleotide at the end of the	This study

	repeat1 AA to GT	
EP-GC	Modified AYEΔF-PA to mutate double-nucleotide at the end of the repeat1 AA to GC	This study
EP-GG	Modified AYEΔF-PA to mutate double-nucleotide at the end of the repeat1 AA to GG	This study
EA-L120	Modified AYEΔF-PA to truncate the leader 131bp to 120 bp	This study
EA-L110	Modified AYEΔF-PA to truncate the leader 131bp to 110 bp	This study
EA-L100	Modified AYEΔF-PA to truncate the leader 131bp to 100 bp	This study
EA-L80	Modified AYEΔF-PA to truncate the leader 131bp to 80 bp	This study
EA-L60	Modified AYEΔF-PA to truncate the leader 131bp to 60 bp	This study
EA-L1	Modified AYEΔF-PA to mutate 21-26 nucleotides of the leader TTAACA to GGCCAC	This study
EA-L2	Modified ECRI-PA to mutate 27-40 nucleotides of the leader GCTCAATAAAATCA to TAGACCGCCCCGAC	This study
EA-L3	Modified ECRI-PA to mutate 41-56 nucleotides of the leader ATAAGTTACAATAGGT to CGCCTGGCACCGCTTG	This study
EA-L4	Modified ECRI-PA to mutate 57-72	This study

	nucleotides of the leader CTTTTTTGATTGGGTA to AGGGGGGTCGGTTTGC	
EA-L5	Modified ECRI-PA to mutate 76-94 nucleotides of the leader TGCCAAAATCCATGATAAA to GTAACCCCGAACGTCGCCC	This study
EA-L6	Modified ECRI-PA to mutate 96-111 nucleotides of the leader ACTTGTTGTAACTTAT to CAGGTGGCGCCAGGCG	This study
EA-L7	Modified ECRI-PA to mutate 112-117 nucleotides of the leader ATTTTT to CGGGGG	This study
EA-L8	Modified ECRI-PA to mutate 118-124 nucleotides of the leader ACTATA to CAGCGC	This study
EA-L9	Modified ECRI-PA to mutate 124-128 nucleotides of the leader ATTT to CGGG	This study
EA-L10	Modified ECRI-PA to mutate 128-134 nucleotides of the leader TATAGT to GCGCTG	This study
A.*baumannii* Ab31	clinical A.*baumannii* strain	(1)
A.*baumannii* Ab64	clinical A.*baumannii* strain	(1)
A.*baumannii* Ab19	clinical A.*baumannii* strain	(1)
A.*baumannii* Ab13	clinical A.*baumannii* strain	(1)
A.*baumannii* Ab100	clinical A.*baumannii* strain	(1)
A.*baumannii* Ab86	clinical A.*baumannii* strain	(1)
A.*baumannii* Ab65	clinical A.*baumannii* strain	(1)

A.*baumannii* Ab30	clinical A.*baumannii* strain	(1)
A.*baumannii* Ab10	clinical A.*baumannii* strain	(1)
A.*baumannii* Ab22	clinical A.*baumannii* strain	(1)
A.*baumannii* Ab83	clinical A.*baumannii* strain	(1)
A.*baumannii* Ab31F	Δ*pyrF* mutant of A.*baumannii* Ab31	This study
A.*baumannii* Ab64F	Δ*pyrF* mutant of A.*baumannii* Ab64	This study
A.*baumannii* Ab19F	Δ*pyrF* mutant of A.*baumannii* Ab19	This study
A.*baumannii* Ab13F	Δ*pyrF* mutant of A.*baumannii* Ab13	This study
A.*baumannii* Ab100F	Δ*pyrF* mutant of A.*baumannii* Ab100	This study
A.*baumannii* Ab86F	Δ*pyrF* mutant of A.*baumannii* Ab86	This study
A.*baumannii* Ab65F	Δ*pyrF* mutant of A.*baumannii* Ab65	This study
A.*baumannii* Ab30F	Δ*pyrF* mutant of A.*baumannii* Ab30	This study
A.*baumannii* Ab10F	Δ*pyrF* mutant of A.*baumannii* Ab10	This study
A.*baumannii* Ab22F	Δ*pyrF* mutant of A.*baumannii* Ab22	This study
A.*baumannii* Ab83F	Δ*pyrF* mutant of A.*baumannii* Ab83	This study
Plasmids		
pMo130Tel^R	Suicide plasmid, xylE, *sacB*+, KmR, Tel^R, ori, oriT.	(2)

pWH1266	Replicative plasmid	(3)
pMo130Tel^R- ΔF	pMo130Tel^R carrying 500bp-flanking fragment of AYE *pyrF*	This study
pMo130TF	Suicide plasmid, replaced *sacB* gene of pMo130Tel^R with *pyrF* gene of AYE	This study
pMo130TFR	replicative plasmid, introduced rep gene of PWH1266 into pMo130TF	This study
pMo130F	Suicide plasmid, replaced *sacB* gene of pMo130 with *pyrF* gene of AYE	This study
pMo130FR	replicative plasmid, introduced rep gene of PWH1266 into pMo130F	This study
pMo130TFRG-P_{tac}	pMo130TFR carrying the tac promoter region and the GFP-mut3 with a strong bacterial RBS-AAAGAGGAGAAA gene	This study
pMo130TFRI	pMo130TFRG-P_{tac} carrying the LacI gene and LacO	This study
pMo130TFR-ECC-sp1	pMo130TFR carrying ECC-sp1 fragment	This study
pMo130TF-ΔCas1	pMo130TF carrying flanking fragment of AYE Cas1	This study
pMo130TF-ΔCas3	pMo130TF carrying flanking fragment of AYE Cas3	This study
pMo130TF-ΔCascade	pMo130TF carrying flanking fragment of AYE Cascade	This study
pMo130TF-aCRISPR	pMo130TF carrying flanking fragment of AYE CRISPR except	This study

	original leader and a single repeat structure	
pMo130 Tel^R-ΔCas1	pMo130Tel^R carrying flanking fragment of AYE Cas1	This study
pMo130 Tel^R-ΔCas3	pMo130Tel^R carrying flanking fragment of AYE Cas3	This study
pMo130 Tel^R-ΔCascade	pMo130Tel^R carrying flanking fragment of AYE Cascade	This study
pMo130 Tel^R-aCRISPR	pMo130TelR carrying flanking fragment of AYE CRISPR except original leader and a single repeat structure	This study
pMo130TFR-EAA-sp1	pMo130TFR carrying protospacer1 of AYE preceded by AA	This study
pMo130TFR-EAT-sp1	pMo130TFR carrying protospacer1 of AYE preceded by AT	This study
pMo130TFR-EAC-sp1	pMo130TFR carrying protospacer1 of AYE preceded by AC	This study
pMo130TFR-EAG-sp1	pMo130TFR carrying protospacer1 of AYE preceded by AG	This study
pMo130TFR-ETA-sp1	pMo130TFR carrying protospacer1 of AYE preceded by TA	This study
pMo130TFR-ETT-sp1	pMo130TFR carrying protospacer1 of AYE preceded by	This study

	TT	
pMo130TFR-ETC-sp1	pMo130TFR carrying protospacer1 of AYE preceded by TC	This study
pMo130TFR-ETG-sp1	pMo130TFR carrying protospacer1 of AYE preceded by TG	This study
pMo130TFR-ECA-sp1	pMo130TFR carrying protospacer1 of AYE preceded by CA	This study
pMo130TFR-ECT-sp1	pMo130TFR carrying protospacer1 of AYE preceded by CT	This study
pMo130TFR-ECC-sp1	pMo130TFR carrying protospacer1 of AYE preceded by CC	This study
pMo130TFR-ECG-sp1	pMo130TFR carrying protospacer1 of AYE preceded by CG	This study
pMo130TFR-EGA-sp1	pMo130TFR carrying protospacer1 of AYE preceded by GA	This study
pMo130TFR-EGT-sp1	pMo130TFR carrying protospacer1 of AYE preceded by GT	This study
pMo130TFR-EGC-sp1	pMo130TFR carrying protospacer1 of AYE preceded by GC	This study

pMo130TFR-EGG-sp1	pMo130TFR carrying protospacer1 of AYE preceded by GG	This study
pMo130TFR-EAA-sp1	pMo130TFR carrying protospacer1 of 19606 preceded by AA	This study
pMo130TFR-EAT-sp1	pMo130TFR carrying protospacer1 of 19606 preceded by AT	This study
pMo130TFR-EAC-sp1	pMo130TFR carrying protospacer1 of 19606 preceded by AC	This study
pMo130TFR-EAG-sp1	pMo130TFR carrying protospacer1 of 19606 preceded by AG	This study
pMo130TFR-ETA-sp1	pMo130TFR carrying protospacer1 of 19606 preceded by TA	This study
pMo130TFR-ETT-sp1	pMo130TFR carrying protospacer1 of 19606 preceded by TT	This study
pMo130TFR-ETC-sp1	pMo130TFR carrying protospacer1 of 19606 preceded by TC	This study
pMo130TFR-ETG-sp1	pMo130TFR carrying protospacer1 of 19606 preceded by TG	This study
pMo130TFR-ECA-sp1	pMo130TFR carrying protospacer1 of 19606 preceded by	This study

	pMo130TFR carrying protospacer1 of 19606 preceded by CA	
pMo130TFR-ECT-sp1	pMo130TFR carrying protospacer1 of 19606 preceded by CT	This study
pMo130TFR-ECC-sp1	pMo130TFR carrying protospacer1 of 19606 preceded by CC	This study
pMo130TFR-ECG-sp1	pMo130TFR carrying protospacer1 of 19606 preceded by CG	This study
pMo130TFR-EGA-sp1	pMo130TFR carrying protospacer1 of 19606 preceded by GA	This study
pMo130TFR-EGT-sp1	pMo130TFR carrying protospacer1 of 19606 preceded by GT	This study
pMo130TFR-EGC-sp1	pMo130TFR carrying protospacer1 of 19606 preceded by GC	This study
pMo130TFR-EGG-sp1	pMo130TFR carrying protospacer1 of 19606 preceded by GG	This study
pMo130TF-pCRISPR	pMo130TF carrying priming CRISPR and chromosomal sequence upstream and downstream of the wild-type CRISPR	This study
pMo130TF-aCRISPR	pMo130TF carrying adaptation CRISPR and chromosomal	This study

	sequences immediately upstream of Cas1 and immediately downstream of fragments of the ABAYE0994 gene	
pMo130TF-pCRISPR-AT	Modified pMo130TF-pCRISPR to mutate double-nucleotide at the end of the repeat1 AA to AT	This study
pMo130TF-pCRISPR-AC	Modified pMo130TF-pCRISPR to mutate double-nucleotide at the end of the repeat1 AA to AC	This study
pMo130TF-pCRISPR-AG	Modified pMo130TF-pCRISPR to mutate double-nucleotide at the end of the repeat1 AA to AG	This study
pMo130TF-pCRISPR-TA	Modified pMo130TF-pCRISPR to mutate double-nucleotide at the end of the repeat1 AA to TA	This study
pMo130TF-pCRISPR-TT	Modified pMo130TF-pCRISPR to mutate double-nucleotide at the end of the repeat1 AA to TT	This study
pMo130TF-pCRISPR-TC	Modified pMo130TF-pCRISPR to mutate double-nucleotide at the end of the repeat1 AA to TC	This study
pMo130TF-pCRISPR-TG	Modified pMo130TF-pCRISPR to mutate double-nucleotide at the end of the repeat1 AA to TG	This study
pMo130TF-pCRISPR-CA	Modified pMo130TF-pCRISPR to mutate double-nucleotide at the end of the repeat1 AA to CA	This study
pMo130TF-pCRISPR-CT	Modified pMo130TF-pCRISPR to	This study

	mutate double-nucleotide at the end of the repeat1 AA to CT	
pMo130TF-pCRISPR-CC	Modified pMo130TF-pCRISPR to mutate double-nucleotide at the end of the repeat1 AA to CC	This study
pMo130TF-pCRISPR-CG	Modified pMo130TF-pCRISPR to mutate double-nucleotide at the end of the repeat1 AA to CG	This study
pMo130TF-pCRISPR-GA	Modified pMo130TF-pCRISPR to mutate double-nucleotide at the end of the repeat1 AA to GA	This study
pMo130TF-pCRISPR-GT	Modified pMo130TF-pCRISPR to mutate double-nucleotide at the end of the repeat1 AA to GT	This study
pMo130TF-pCRISPR-GC	Modified pMo130TF-pCRISPR to mutate double-nucleotide at the end of the repeat1 AA to GC	This study
pMo130TF-pCRISPR-GG	Modified pMo130TF-pCRISPR to mutate double-nucleotide at the end of the repeat1 AA to GG	This study
pMo130TF-aCRISPR-L120	Modified pMo130TF-aCRISPR to truncate the leader 131bp to 120 bp	This study
pMo130TF-aCRISPR-L110	Modified pMo130TF-aCRISPR to truncate the leader 131bp to 110 bp	This study
pMo130TF-aCRISPR-L100	Modified pMo130TF-aCRISPR to truncate the leader 131bp to 100 bp	This study
pMo130TF-aCRISPR-L80	Modified pMo130TF-aCRISPR to truncate the leader 131bp to 80 bp	This study
pMo130TF-aCRISPR-L60	Modified pMo130TF-aCRISPR to	This study

	truncate the leader 131bp to 60 bp	
pMo130TF-aCRISPR-L1	Modified pMo130TF-aCRISPR to mutate 21-26 nucleotides of the leader TTAACA to GGCCAC	This study
pMo130TF-aCRISPR-L2	Modified pMo130TF-aCRISPR to mutate 27-40 nucleotides of the leader GCTCAATAAAATCA to TAGACCGCCCCGAC	This study
pMo130TF-aCRISPR-L3	Modified pMo130TF-aCRISPR to mutate 41-56 nucleotides of the leader ATAAGTTACAATAGGT to CGCCTGGCACCGCTTG	This study
pMo130TF-aCRISPR-L4	Modified pMo130TF-aCRISPR to mutate 57-72 nucleotides of the leader CTTTTTTGATTGGGTA to AGGGGGGTCGGTTTGC	This study
pMo130TF-aCRISPR-L5	Modified pMo130TF-aCRISPR to mutate 76-94 nucleotides of the leader TGCCAAAATCCATGATAAA to GTAACCCCGAACGTCGCCC	This study
pMo130TF-aCRISPR-L6	Modified pMo130TF-aCRISPR to mutate 96-111 nucleotides of the leader ACTTGTTGTAACTTAT to CAGGTGGCGCCAGGCG	This study
pMo130TF-aCRISPR-L7	Modified pMo130TF-aCRISPR to mutate 112-117 nucleotides of the leader ATTTTT to CGGGGG	This study

pMo130TF-aCRISPR-L8	Modified pMo130TF-aCRISPR to mutate 118-124 nucleotides of the leader ACTATA to CAGCGC	This study
pMo130TF-aCRISPR-L9	Modified pMo130TF-aCRISPR to mutate 124-128 nucleotides of the leader ATTT to CGGG	This study
pMo130TF-aCRISPR-L10	Modified pMo130TF-aCRISPR to mutate 128-134 nucleotides of the leader TATAGT to GCGCTG	This study

TABLE S2 The primers used in this study.

Primer	Sequence(5′→3′)
Δ*pyrF*-UF	GCATGCATCTAGAGGGATCCGTGGGGTGCCTATGCGTTAT
Δ*pyrF* -UR	GCACATAAAATCAGCGCTA
Δ*pyrF* -DF	GTAGCGCTGATTTTATGTG TGCTTCAATTGCTTAAAA
Δ*pyrF* -DR	CCTGAGCGGCCGCCCTGCAG GGCCAAGGCATTTTTAGCT
Kana-F	CCTGAGCGGCCGCCCTGCAGCGGATCC
Kana-R	TCAGAAGAACTCGTCAAGAA
pyrF -F	TTCTTGACGAGTTCTTCTGAGGTACCTTTTGGTACTACTCTTTATCT
pyrF -R	GCTAACTTACATTAATTGCGGAATTCTTAAGCAATTGAAGCAAGAA
Rep-F	TTCTTGCTTCAATTGCTTAAGAATTCGGGGATCGTAGAAATAT
Rep-R	GCTAACTTACATTAATTGCGGAATTCGGGGGATTTTAACATTTTGC
ΔCas1-UF	GCATGCATCTAGAGGGATCCCAGCCAGAGTTTGAATTACCTAT
ΔCas1-UR	TACAGATTAGCTCGTTTAGA
ΔCas1-DF	TCTAAACGAGCTAATCTGTACGACTGAGCAGGAGTTCCGT
ΔCas1-DR	CCTGAGCGGCCGCCCTGCAG TTCCTCGTCCCAAGTTTGATTC
ΔCas3-UF	GCATGCATCTAGAGGGATCCTTGCCCGGATTGAATTTAT
ΔCas3-UR	CGCGACGAGTTCGAGGAATC
ΔCas3-DF	GATTCCTCGAACTCGTCGCGGTCGTTTAGCGCTTGATAAA
ΔCas3-DR	CCTGAGCGGCCGCCCTGCAG TTCGAAACCATCCAATACTGTCA
ΔCascade-UF	GCATGCATCTAGAGGGATCCATGGCAGATGCTTTAACTTCA
ΔCascade-UR	TCATTTAAAAATGTATGGAT
ΔCascade-DF	ATCCATACATTTTTAAATGAGGACTAAGCCGAATGACAAC
ΔCascade-DR	CCTGAGCGGCCGCCCTGCAGGCCCGTAGGTGGTCGATCTGTAACA
aCRISPR-UF	GCATGCATCTAGAGGGATCCTGCCAGCTCGGAAAATGACTTACAA

aCRISPR-UR	AACCATTTCTAAATGGCGT
aCRISPR-DF	ACGCCATTTAGAAATGGTTATGACGGTATACGTCACT
aCRISPR-DR	CCTGAGCGGCCGCCCTGCAGCCGCACCAAAGGCAACATAAGT
Tac-GFP-F1-PstI	CCTGAGCGGCCGCCCTGCAGGAGCTGTTGACAATTAATCATCGG CTCGTATAATGTGTG
Tac-GFP-F2	CGGCTCGTATAATGTGTGGAATTGTGAGCGGATAACAATTTCACA CAGGAAACAGAATT
Tac-GFP-F3	CAATTTCACACAGGAAACAGAATTCTATGCGTAAAGGAGAAGAA
Tac-GFP-R-BamHI	GCATGCATCTAGAGGGATCCTTCTTCTCCTTTACGCAT
LacI-F-BamHI	TGGATGAACTATACAAATAAGGATCCGACACCATCGAATGGTGCA A
LacI-R-sphI	CAGACAAGCCCGTCGCATGCTCACTGCCCGCTTTCCAG
pMo130TFR-ECC-sp1-F	GCATGCATCTAGAGGGATCCCCTGGTTGGGCATTAATCAGGT
pMo130TFR-ENN-sp1-R	CGTGCTGACCTGACCTGAGCGGCCGCAATTAAAAACCCACCTGA TT
pMo130TFR-EAA-sp1-F	GCATGCATCTAGAGGGATCCAATGGTTGGGCATTAATCAGGT
pMo130TFR-EAT-sp1-F	GCATGCATCTAGAGGGATCCATTGGTTGGGCATTAATCAGGT
pMo130TFR-EAC-sp1-F	GCATGCATCTAGAGGGATCCACTGGTTGGGCATTAATCAGGT
pMo130TFR-EAG-sp1-F	GCATGCATCTAGAGGGATCCAGTGGTTGGGCATTAATCAGGT
pMo130TFR-ETA-sp1-F	GCATGCATCTAGAGGGATCCTATGGTTGGGCATTAATCAGGT
pMo130TFR-ETT-sp1-F	GCATGCATCTAGAGGGATCCTTTGGTTGGGCATTAATCAGGT
pMo130TFR-ETC-sp1-F	GCATGCATCTAGAGGGATCCTCTGGTTGGGCATTAATCAGGT
pMo130TFR-ETG-	GCATGCATCTAGAGGGATCCTGTGGTTGGGCATTAATCAGGT

sp1-F	
pMo130TFR-ECA-sp1-F	GCATGCATCTAGAGGGATCCCATGGTTGGGCATTAATCAGGT
pMo130TFR-ECT-sp1-F	GCATGCATCTAGAGGGATCCCTTGGTTGGGCATTAATCAGGT
pMo130TFR-ECG-sp1-F	GCATGCATCTAGAGGGATCCCGTGGTTGGGCATTAATCAGGT
pMo130TFR-EGA-sp1-F	GCATGCATCTAGAGGGATCCGATGGTTGGGCATTAATCAGGT
pMo130TFR-EGT-sp1-F	GCATGCATCTAGAGGGATCCGTTGGTTGGGCATTAATCAGGT
pMo130TFR-EGC-sp1-F	GCATGCATCTAGAGGGATCCGCTGGTTGGGCATTAATCAGGT
pMo130TFR-EGG-sp1-F	GCATGCATCTAGAGGGATCCGGTGGTTGGGCATTAATCAGGT
EXF	AAACAATATGTTGAGCAA
EXR	AATTAAAAACCCACCTGATT
EACRI-F	TTGAGCAACCAGTGGTTGAA
EACRI-R	GCACCAAAGGCAACATAAGT
EPCRI-F	GATAAGATTGATTCTTAA
EPCRI-R	TTTATCATATTCTCTCTT
p130-F	GCGGGGGAGATTACAACTAC
p130-R	GCGCGTTTCGGTGATGA
pMo130TF-pCRISPR-FPF-SphI	ACATGCATGCTGCCGTGTCATCTGCTTCAAAAAC
pMo130TF-pCRISPR-FPR-BamHI	CGCGGATCCTTAAGAATCAATCTTATC

pMo130TF-pCRISPR-PPF-PstI	AAAACTGCAG AAGAGAGAATATGATAAA
pMo130TF-pCRISPR-PPR-NotI	ATAAGAATGCGGCCGCCGCCCCTTCTTTAGCACAA
pMo130TF-pCRISPR-F1-BamHI	CGCGGATCC AACGCTCGAATTAATGGATGT
pMo130TF-pCRISPR-R1-PstI	AAAACTGCAGGCGCAAAACCTTGTTGATTAT
pMo130TF-aCRISPR-FPF-SphI	ACATGCATGCTGCGGAGAAAGTAGAAGTTGTTGTAA
pMo130TF-aCRISPR-FPR-BamHI	CGCGGATCC TGAGCTGTTAAAGAATAGA
pMo130TF-aCRISPR-PPF-PstI	AAAACTGCAGATGACGGTATACGTCACTTA
pMo130TF-aCRISPR-PPR-NotI	ATAAGAATGCGGCCGCCCGCACCAAAGGCAACATAAGT
pMo130TF-aCRISPR-F1-BamHI	CGCGGATCCCAATATTTTTTCTATTCTTTAACAGCTCAATAAAATC AATAAGTTACAA
pMo130TF-aCRISPR-R1-PstI	AAAACTGCAGTTTCTAAATGGCGTATGCCGCCATGAAC
pMo130TF-aCRISPR-Leader-R2	GTGACGTATACCGTCATCTGCAGAACCTTGTTGATTATATC

pMo130TF-aCRISPR-L1-F	CCCAATATTTTTTCTATTCTGGCCACGCTCAATAAAATCAA
pMo130TF-aCRISPR-L1-R	TTGATTTTATTGAGCGTGGCCAGAATAGAAAAAATATTGGG
pMo130TF-aCRISPR-L2-F	TAGACCGCCCCGACATAAGTTACAATAGG
pMo130TF-aCRISPR-L2-R	ATGTCGGGGCGGTCTATGTTAAAGAATAGAA
pMo130TF-aCRISPR-L3-F	CGCCTGGCACCGCTTGCTTTTTTGATTGGGT
pMo130TF-aCRISPR-L3-R	CAAGCGGTGCCAGGCGTGATTTTATTGAGCT
pMo130TF-aCRISPR-L4-F	AGGGGGGTCGGTTTGCAAATGCCAAAATCCA
pMo130TF-aCRISPR-L4-R	GCAAACCGACCCCCCTACCTATTGTAACTTA
pMo130TF-aCRISPR-L5-F	GTAACCCCGAACGTCGCCCCACTTGTTGTAACTT
pMo130TF-aCRISPR-L5-R	GGGCGACGTTCGGGGTTACTTTTACCCAATCAAA
pMo130TF-aCRISPR-L6-F	CAGGTGGCGCCAGGCGATTTTTACTATAATT
pMo130TF-aCRISPR-L6-R	CGCCTGGCGCCACCTGGTTTATCATGGATTTTG
pMo130TF-aCRISPR-L7-F	CGGGGGACTATAATTTTATAGT
pMo130TF-aCRISPR-L7-R	ACTATAAAATTATAGTCCCCCGATAAGTTACAACAAG
pMo130TF-	CAGCGCATTTTATAGTTCATG

aCRISPR-L8-F	
pMo130TF-aCRISPR-L8-R	CATGAACTATAAAATGCGCTGAAAAATATAAGTTAC
pMo130TF-aCRISPR-L9-F	CGGGTATAGTTCATGGCGG
pMo130TF-aCRISPR-L9-R	CCGCCATGAACTATACCCGTATAGTAAAAATATA
pMo130TF-aCRISPR-L10-F	GCGCTGTCATGGCGGCATACG
pMo130TF-aCRISPR-L10-R	CGTATGCCGCCATGACAGCGCAAATTATAGTAAAAAT
pMo130TF-aCRISPR-L60-R-BamHI	CGCGGATCCAAAATGCCAAAATCCATGATAAACACTTGTTGTAACTTAT
pMo130TF-aCRISPR-L80-R-BamHI	CGCGGATCCTAGGTCTTTTTTGATTGGGTAAAATGCCAAAATCCATGAT
pMo130TF-aCRISPR-L100-R-BamHI	CGCGGATCCATAAAATCAATAAGTTACAATAGGTCTTTTTTGATTGGGT
pMo130TF-aCRISPR-L110-R-BamHI	CGCGGATCCTAACAGCTCAATAAA
pMo130TF-aCRISPR-L120-R-BamHI	CGCGGATCCTTCTATTCTTTAACAGCTCA
pMo130TF-aCRISPR-L131-R-BamhI	CGCGGATCCCAATATTTTTTCTATTCTTTAACAGCTCAATAAAATCAATAAGTTACAA

Reference

1. Liu C, Chang Y, Xu Y, Luo Y, Wu L, Mei Z, Li S, Wang R, Jia X. 2018. Distribution of virulence-associated genes and antimicrobial susceptibility in clinical Acinetobacter baumannii isolates. Oncotarget 9:21663-21673.
2. Amin I, Richmond G, Sen P, Koh T, Piddock L, Chua K. 2013. A method for generating marker-less gene deletions in multidrug-resistant Acinetobacter baumannii. BMC microbiology 13:158.
3. Hunger M, Schmucker R, Kishan V, Hillen W. 1990. Analysis and nucleotide sequence of an origin of DNA replication in Acinetobacter calcoaceticus and its use for Escherichia coli shuttle plasmids. Gene 87:45-51.
